# Tuning the Properties of Donor–Acceptor and Acceptor–Donor–Acceptor Boron Difluoride Hydrazones *via* Extended π-Conjugation

**DOI:** 10.1021/acsomega.2c04401

**Published:** 2022-08-26

**Authors:** Daniela Cappello, Francis L. Buguis, Joe B. Gilroy

**Affiliations:** Department of Chemistry and The Centre for Advanced Materials and Biomaterials Research (CAMBR), The University of Western Ontario, London, Ontario N6A 5B7, Canada

## Abstract

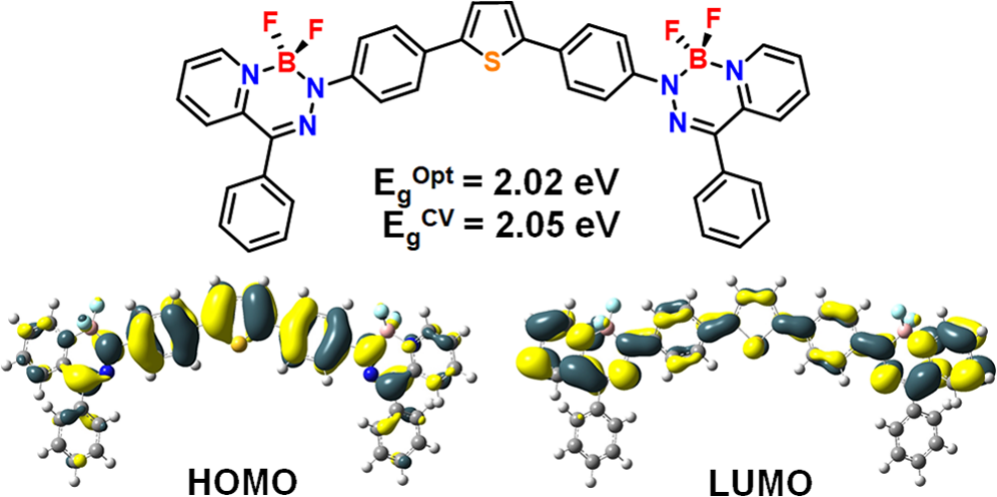

Molecular materials with π-conjugated donor–acceptor (D–A) and acceptor–donor–acceptor (A–D–A) electronic structures have received significant attention due to their usage in organic photovoltaic materials, in organic light-emitting diodes, and as biological imaging agents. Boron-containing molecular materials have been explored as electron-accepting units in compounds with D–A and A–D–A properties as they often exhibit unique and tunable optoelectronic and redox properties. Here, we utilize Stille cross-coupling chemistry to prepare a series of compounds with boron difluoride hydrazones (BODIHYs) as acceptors and benzene, thiophene, or 9,9-dihexylfluorene as donors. BODIHYs with D–A and A–D–A properties exhibited multiple reversible redox waves, solid-state emission with photoluminescence quantum yields up to 10%, and aggregation-induced emission (AIE). Optical band gaps (or highest occupied molecular orbital (HOMO)–lowest unoccupied molecular orbital (LUMO) gaps) determined for these compounds (2.02–2.25 eV) agree well with those determined from cyclic voltammetry experiments (2.05–2.42 eV). The optoelectronic properties described herein are rationalized with density functional theory calculations that support the interpretation of the experimental findings. This work provides a foundation of understanding that will allow for the consideration of D–A and A–D–A BODIHYs to be incorporated into applications (*e.g.*, organic electronics) where fine-tuning of band gaps is required.

## Introduction

**Figure 1:**
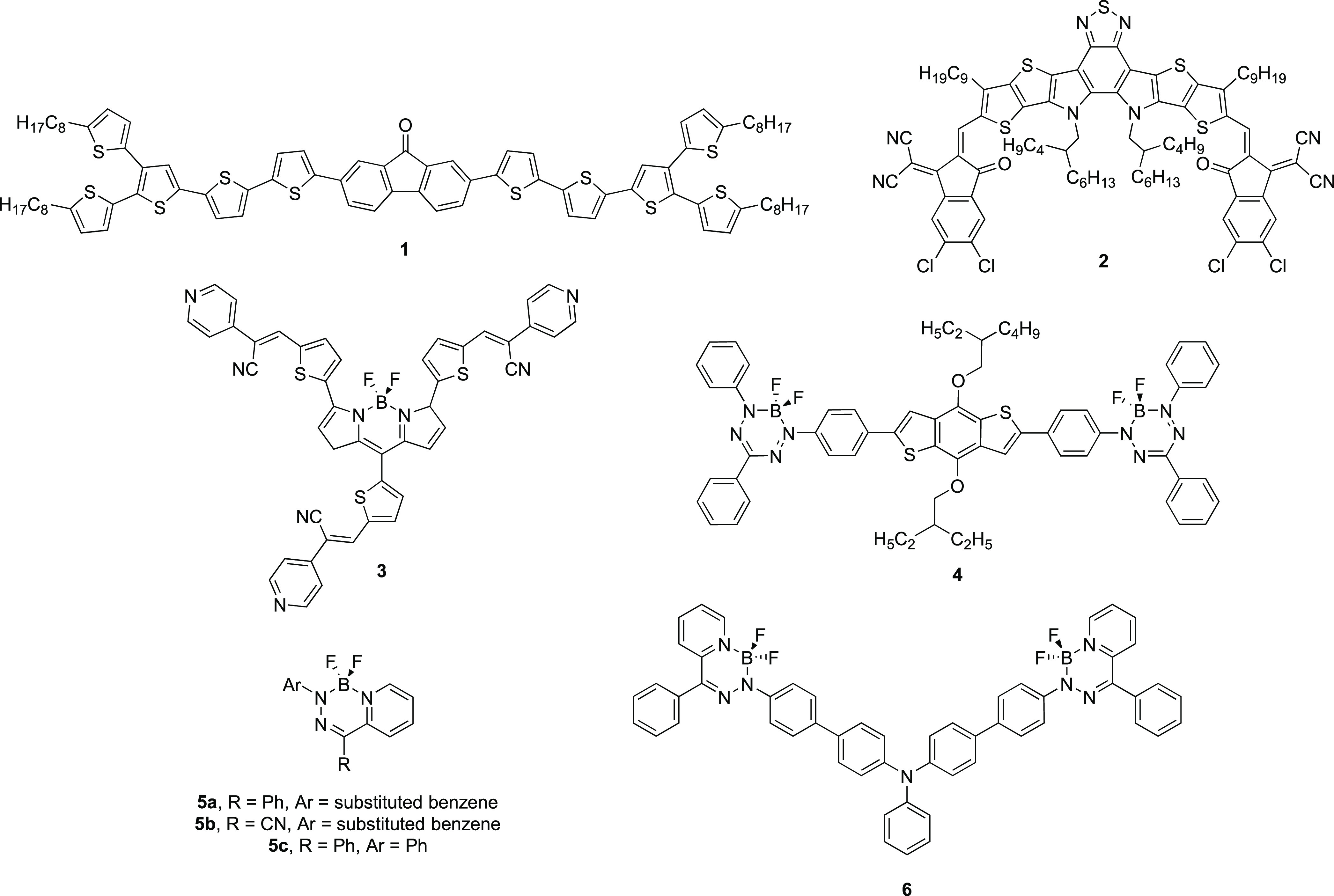
Representative donor–acceptor, acceptor–donor–acceptor, and boron difluoride hydrazone (BODIHY) compounds.

Donor–acceptor (D–A) and acceptor–donor–acceptor (A–D–A) molecules that benefit from extended π-conjugation have been widely studied as a result of their unique properties such as charge transfer,^1−10^ thermally activated delayed fluorescence,^5,11−18^ and aggregation-induced emission (AIE) (Figure 1).^19−26^ This has led to their exploitation in a number of applications including organic light-emitting diodes,^26−29^ organic photovoltaic materials,^30−47^ and bioimaging.^13,48−52^ In order for molecular materials to be utilized in the aforementioned applications, control over the highest occupied molecular orbital (HOMO) and lowest unoccupied molecular orbital (LUMO) energies is often required. Incorporating heteroatoms (*e.g.*, N, O, S, B) into π-conjugated systems is one potential avenue for the alteration of optoelectronic properties, such as the band gap (E_g_). When **1**, which contains several thiophene (TH) groups appended to a fluorenone core, was incorporated into bulk heterojunction solar cells the power conversion efficiency (PCE) reached up to 0.8%.^53^ When **2** was incorporated into an organic solar cell, a PCE of 18% was achieved, which is the highest PCE reported to date for solar cells employing nonfullerene acceptors.^54^

Alternatively, electron-deficient boron-containing compounds have been integrated into D–A and A–D–A compounds as the electron acceptor component. Heterocycles based on boron difluoride (BF_2_) adducts of chelating *N*-donor ligands are molecular materials that offer useful and potentially tunable redox, absorption, and emission properties. Perhaps the most well-known of these materials are the boron dipyrromethenes (BODIPYs), which have been highly sought after due to their narrow absorption and emission profiles, very high emission quantum yields (Φ_em_) in solution, and rich redox chemistry.^55−57^ BODIPYs have been incorporated into molecular materials with an A–D–A architecture (*e.g.*, **3**) and exhibit near-infrared absorption and emission, electrochemical band gaps (E_g_^CV^) as low as 1.67 eV, and the ability to generate singlet oxygen.^58^ Combining TH donors with BF_2_-containing materials, such as BODIPYs, has resulted in red-shifted emission wavelengths, sometimes into the near-infrared region, due to the expansion of the π-electron system.^59^ Our group has recently demonstrated that when BF_2_ formazanates are bridged by a strong donor such as benzodithiophene (**4**), an E_g_^CV^ of 1.21 eV and an optical band gap (E_g_^Opt^) of 1.38 eV can be achieved.^60^

The purpose of this work was to incorporate boron difluoride hydrazones (BODIHYs) as the electron acceptors in D–A and A–D–A scaffolds to demonstrate the ease of property tuning for these unique molecular materials *via* extended π-conjugation. BODIHYs (*e.g.*, **5**) are BF_2_-containing heterocycles based on chelating pyridylhydrazone ligands, and these materials exhibit aggregation-induced emission and interesting redox chemistry.^61−68^ We recently developed triphenylamine–BODIHY conjugates (*e.g.*, **6**) that exhibit dual emission, AIE, multiple redox waves, and charge-transfer characteristics.^61^ Inspired by the traits associated with these BODIHY-bearing strong triarylamine donors, we were interested in utilizing BODIHYs as electron acceptors and altering the nature of the donor in D–A and A–D–A architectures. Herein, we present a systematic approach to investigating structure–property relationships for BODIHYs linked to several donors in D–A and A–D–A molecular frameworks and present their optoelectronic, AIE, and redox properties.

## Results and Discussion

### Synthesis

**Scheme 1:**
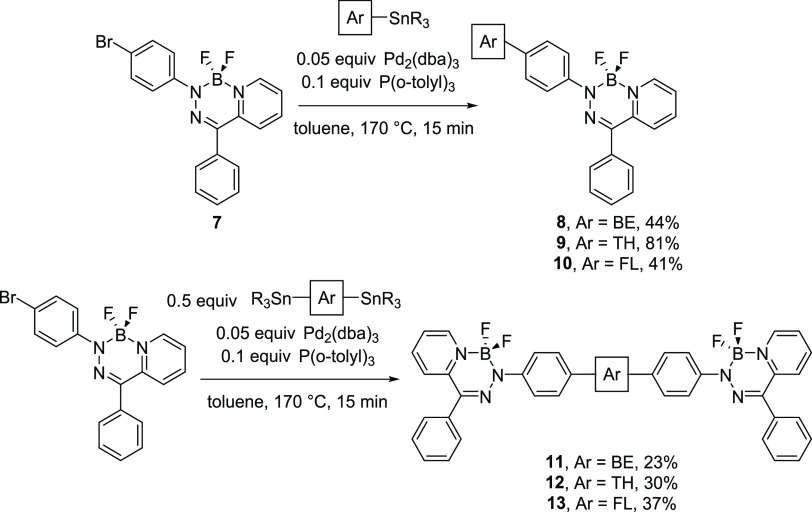
Synthetic Pathways for the Production of D–A and A–D–A BODIHYs **8**–**13**

We utilized Stille cross-coupling reactions for the production of D–A and A–D–A BODIHYs by modifying a procedure used to prepare a related family of compounds (Scheme 1).^60^ Our approach began by preparing Br–BODIHY **7** according to a literature procedure.^61^ Benzene (BE), thiophene (TH), and 9,9-dihexylfluorene (FL) were chosen as donors due to the commercial availability and/or synthetic accessibility of the relevant synthons (Figures S1 and S2) and the variation of the size of the corresponding π-electron systems. BODIHY **7** was combined with monosubstituted organotin reagents of BE, TH, or FL using 5 mol % Pd_2_(dba)_3_/10 mol % P(*o*-tolyl)_3_ in toluene and heated to 170 °C with stirring for 15 min in a sealed tube (pressure 3.2–4.3 bar) to yield BODIHYs **8**–**10** (Figures S3–S8). BODIHYs **11**–**13** were prepared similarly using disubstituted organotin reagents of BE, TH, and FL (Figures S9–S14). To purify BODIHYs **8**–**13**, we used column chromatography (silica gel; 3:1 CH_2_Cl_2_/hexane), which resulted in their isolation in yields ranging from 23 to 81%. Despite several attempts, we were unable to grow single crystals of the title compounds, which precluded X-ray crystallography studies. To probe the thermal stability of compounds **8**–**10**, **12**, and **13**, thermal gravimetric analysis experiments were conducted, and it was found that the title compounds were thermally stable (*i.e.*, retained 98% of their mass) up to a minimum of 190 °C with thermal stability windows extending to maximum values of nearly 270 °C for select compounds (Figure S15).

### Absorption and Emission Properties

**Figure 2:**
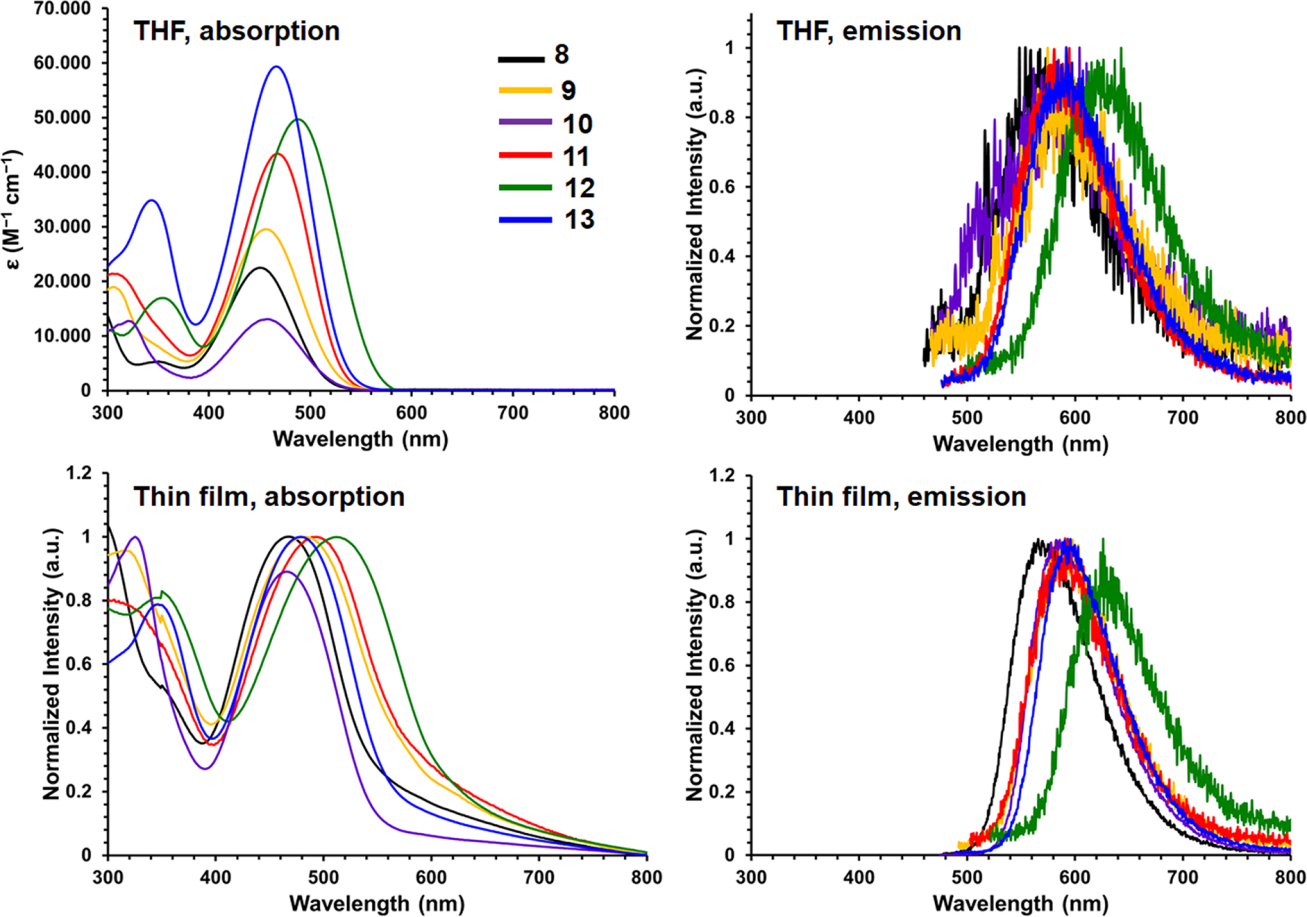
UV–vis absorption and emission spectra of BODIHYs **8**–**13**.

The absorption and emission properties of BODIHYs **8**–**13** were explored experimentally using ultraviolet–visible (UV–vis) spectroscopy (Figure 2 and Table 1) and will be discussed in comparison to the properties of BODIHY **5c**, which possesses phenyl substituents.^62^ The wavelengths of maximum absorption (λ_max_) of BODIHYs with extended π-electron systems were red-shifted relative to **5c** (λ_max_ = 437 nm in tetrahydrofuran (THF)): Δλ_max_ = 13 nm for **8**, Δλ_max_ = 19 nm for **9**, Δλ_max_ = 20 nm for **10**, Δλ_max_ = 31 nm for **11**, Δλ_max_ = 50 nm for **12**, and Δλ_max_ = 29 nm for **13**. The spectra collected in both CH_2_Cl_2_ and toluene revealed similar trends. The λ_max_ values determined experimentally agreed well with those calculated using time-dependent DFT (TDDFT) (Table S1). Solvatochromism and a decrease in Φ_em_ were observed upon increasing the polarity of the solvent (*i.e.*, toluene → THF), supporting the hypothesis that the relevant electronic transitions observed for BODIHYs **11**–**13** may have charge-transfer character. Like other BODIHYs,^61−63,67,68^ compounds **8–13** exhibited weak solution-state emission due to nonradiative relaxation pathways arising from the rotation and/or vibration of the aryl substituents appended to the BODIHY heterocycle. The wavelength of maximum emission (λ_em_) for BODIHY **5c** in THF is 547 nm and λ_em_ for **8**–**13** were red-shifted by Δλ_em_ = 2 nm for **8**, Δλ_em_ = 29 nm for **9**, Δλ_em_ = 57 nm for **10**, Δλ_em_ = 48 nm for **11**, Δλ_em_ = 96 nm for **12**, and Δλ_em_ = 45 nm for **13**, with Φ_em_ < 1% for all compounds. To explore the solid-state properties of BODIHYs **8**–**13**, we prepared thin films by spin-coating saturated CH_2_Cl_2_ solutions of each BODIHY onto quartz slides and measured the corresponding absorption and emission spectra. The absorption and emission bands observed for the thin films were broadened, λ_max_ and λ_em_ values were red-shifted, and Φ_em_ values ranged from 3 to 10%, which were in some cases enhanced relative to other BODIHYs with extended π-conjugation reported to date.^61,63^ The onset of absorption was used to estimate E_g_^Opt^ using the equation E_g_^Opt^ = 1240/λ_max_^onset^ (see Table 3 below). Relative to BODIHY **5c**, which has an E_g_^Opt^ of 2.45 eV, there was a noticeable decrease in E_g_^Opt^ for compounds **8**–**13** ranging from 2.02 to 2.25 eV, with compound **12** possessing the lowest E_g_^Opt^. Given that BODIHY emission is diminished by the secondary inner-filter effect^61,63^ relating to absorption/emission spectral overlap (Figure S16), we also collected spectra for thin films spin-coated from CH_2_Cl_2_ solutions containing BODIHYs and poly(methyl methacrylate) (PMMA) in a 1:99 ratio (by weight). In doing so, we effectively weakened the reabsorption associated with tailing of the absorption spectra at low energies that overlap with the corresponding emission bands. The emission intensities for these films were enhanced and blue-shifted relative to those observed for films of the pure compounds (Figure S17).

**Table 1:** Absorption and Emission Properties of BODIHYs **5c** and **8**–**13**^a^,^b^,^c^,^d^

		λ_max_ (nm)	ε (M^–1^ cm^–1^)	λ_em_ (nm)	Φ_em_ (%)	υ_ST_ (nm)	υ_ST_ (cm^–1^)
**5c**(62)	THF	437	20,100	547	<1	110	4602
	CH_2_Cl_2_	437	13,200	540	<1	103	4365
	toluene	447	11,600	542	<1	95	3921
	thin film	444		525	18	81	3475
	aggregate	454		567	2	113	4390
**8**	THF	450	21,500	549	<1	99	4007
	CH_2_Cl_2_	452	19,600	585	<1	133	5030
	toluene	462	18,300	559	1	97	3756
	thin film	468		566	4	98	3700
	aggregate	445		574	4	129	5050
**9**	THF	456	28,600	575	<1	119	4539
	CH_2_Cl_2_	458	16,300	594	5	136	4999
	toluene	469	27,000	590	<1	121	4373
	thin film	482		593	3	111	3884
	aggregate	453		591	2	138	5155
**10**	THF	457	12,500	604	<1	147	5326
	CH_2_Cl_2_	458	12,100	591	<1	133	4914
	toluene	467	13,800	579	<1	112	4142
	thin film	465		587	7	122	4470
	aggregate	457		587	4	130	4846
**11**	THF	468	42,800	595	<1	127	4561
	CH_2_Cl_2_	467	41,500	593	<1	126	4545
	toluene	479	8300	582	1	103	3695
	thin film	493		591	10	98	3364
**12**	THF	487	49,900	643	<1	156	4982
	CH_2_Cl_2_	487	45,000	614	1	127	4247
	toluene	502	5300	614	1	112	3634
	thin film	512		626	4	114	4310
	aggregate	506		625	3	119	3763
**13**	THF	466	58,900	592	<1	126	4567
	CH_2_Cl_2_	466	56,900	590	<1	124	4510
	toluene	485	54,800	578	1	93	3576
	thin film	479		596	5	117	4098
	aggregate	466		596	4	130	4681

a Solution spectra were collected for 5 μM analyte solutions in dry and degassed solvents.

b The integrating sphere method was used to determine absolute quantum yields.

c Thin films on quartz slides were spin-coated from CH_2_Cl_2_ solutions.

d The aggregate spectra were collected for the 25 μM THF/H_2_O mixtures of each compound that gave maximum emission intensities.

### Aggregation-Induced Emission

**Figure 3:**
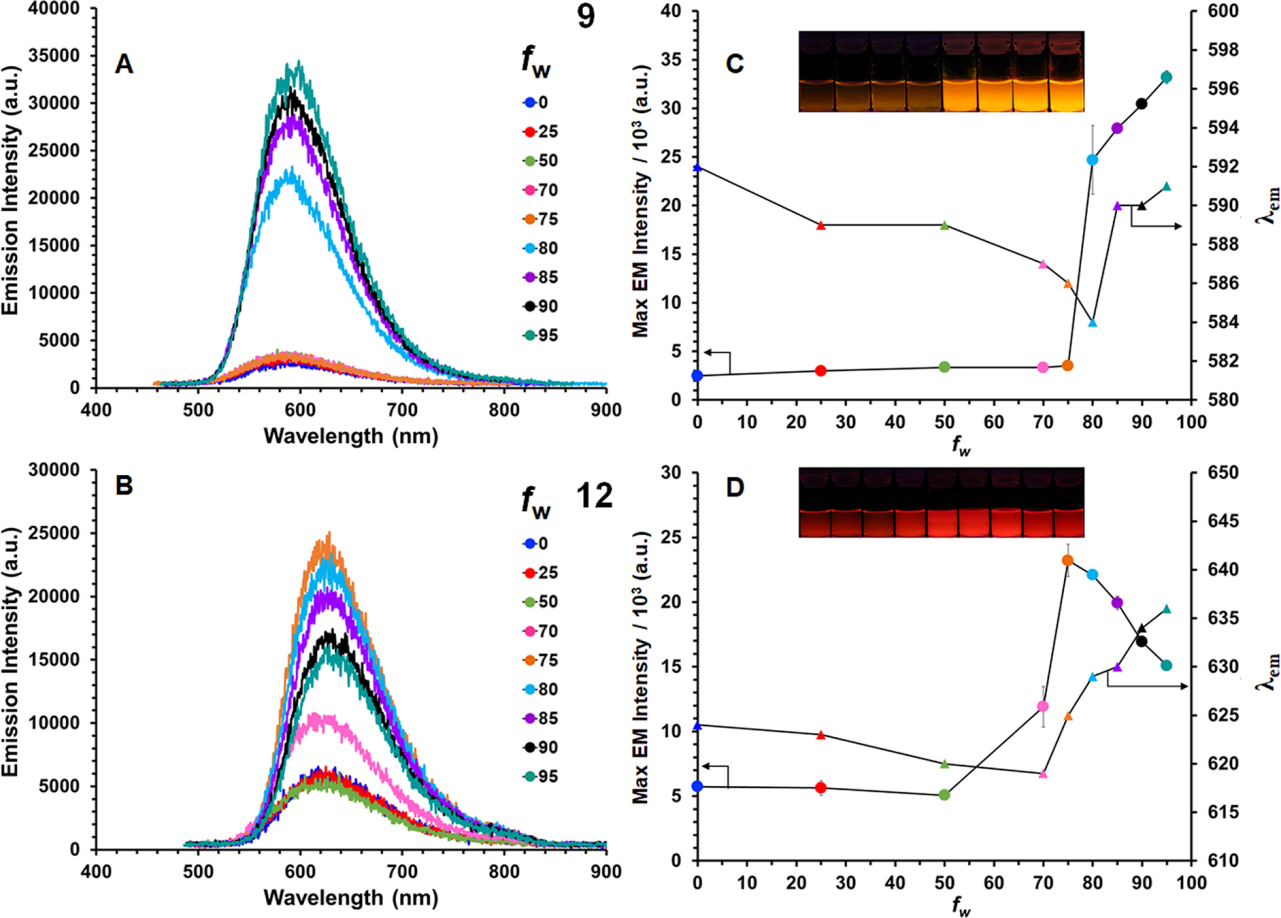
Emission spectra for solutions of **9** (A) and **12** (B) at various *f*_w_ in THF. Maximum emission intensities (circles) and λ_em_ (triangles) for **9** (C) and **12** (D) plotted as a function of *f*_w_ in THF. Concentrations were 25 μM of the analyte. Each data point represents the average from experiments conducted in triplicate.

The AIE of BODIHYs **8–10**, **12**, and **13** was explored by preparing 25 μM solutions of varying H_2_O (*f*_w_) fractions in THF and collecting their emission spectra (Figures 3 and S18–S20). Due to the limited solubility and instantaneous precipitation upon aggregate formation, the AIE behavior of BODIHY **11** was not further examined. An initial blue shift in λ_em_ was observed when H_2_O was added to THF solutions of BODIHYs **8–10**, **12**, and **13** while the emission intensity remained low until *f*_w_ reached 50% for **10** and **13**, 70% for **12**, and 75% for **8** and **9**. Subsequent addition of H_2_O resulted in red-shifted λ_em_ and increased emission intensities where maximum emission intensities were detected for when *f*_w_ = 95% for **8** (Φ_em_ = 4%), *f*_w_ = 95% for **9** (Φ_em_ = 2%), *f*_w_ = 95% for **10** (Φ_em_ = 4%), *f*_w_ = 75% for **12** (Φ_em_ = 3%), and *f*_w_ = 85% for **13** (Φ_em_ = 4%). The increased emission intensity is consistent with restriction of intramolecular motion (RIM) of the aryl substituents upon aggregation that was induced upon the addition of H_2_O. Enhancement factors were calculated from the ratio of maximum emission intensity and the emission intensity measured at *f*_w_ = 0%. They were 14 for **8**, 13 for **9**, 12 for **10**, 4 for **12**, and 9 for **13**. The observed red shift in λ_em_ with increasing *f*_w_ and the blue-shifted and enhanced emission observed for thin films containing PMMA doped with 1% BODIHY are consistent with trends observed for related BODIHYs^61−63^ and imply that RIM is a plausible contributor to the AIE behavior observed.

### Electronic Structure Investigations

**Figure 4:**
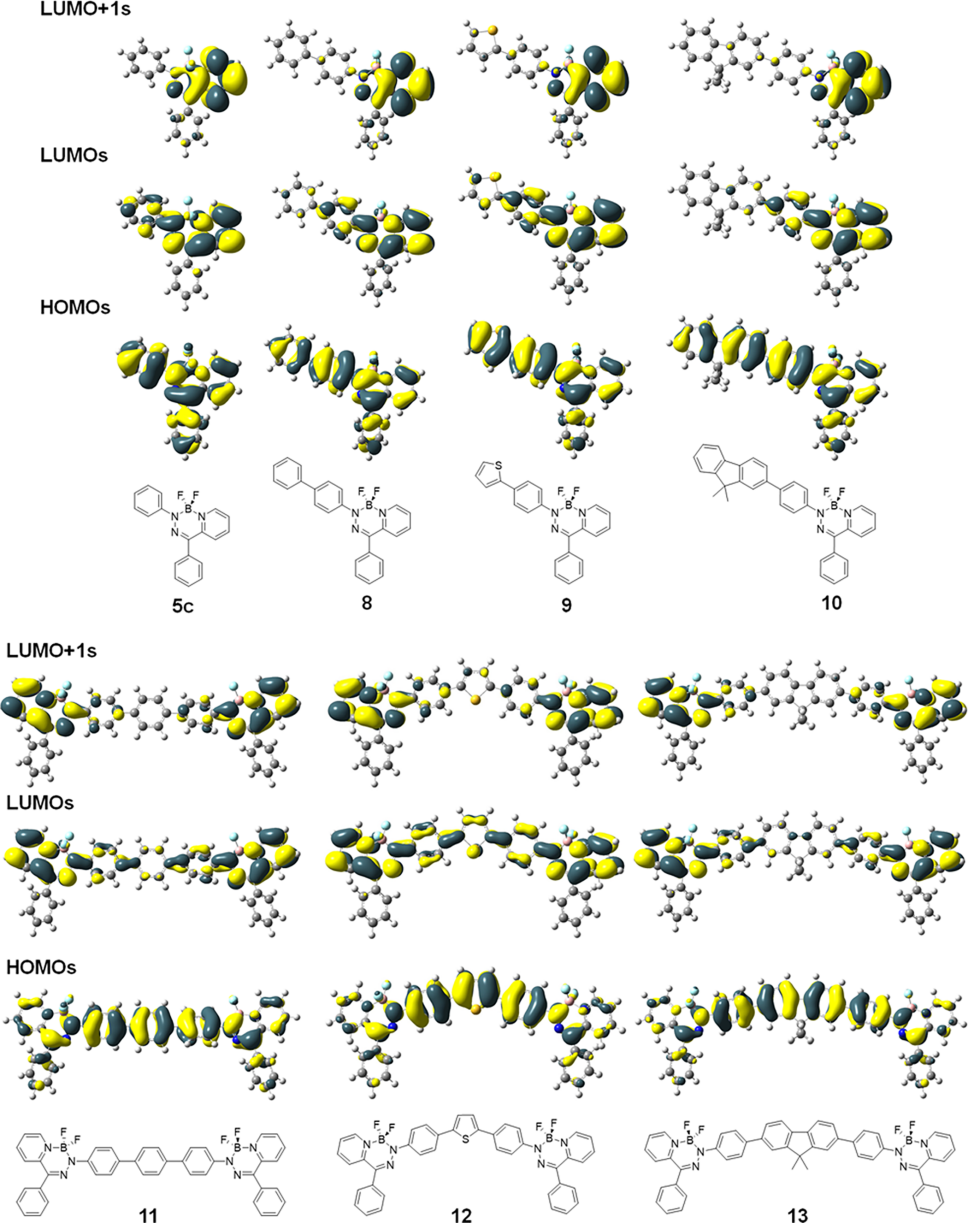
Selected molecular orbitals for BODIHYs **5c** and **8**–**13** (ground state, LC-ωhPBE (ω = 0.14)/DGDZVP2 SCRF = (PCM, solvent = THF) method). The hexyl chains of BODIHYs **10** and **13** were approximated as methyl groups.

The electronic structures of BODIHYs **5c** and **8**–**13** were investigated computationally using density functional theory (DFT; LC-ωhPBE (ω = 0.14)/DGDZVP2)^69,70^ (Figures 4, S21–S26, and Table S1). TDDFT implicated the respective HOMOs, LUMOs, and LUMO + 1s as the orbitals contributing to the low-energy absorption bands for BODIHYs **5c** and **8–13**. In the cases of **5c** and D–A BODIHYs **8**–**10**, the HOMOs and LUMOs were delocalized π-type orbitals, suggesting that the low-energy transitions were of π → π* type. In A–D–A BODIHYs **11**–**13**, the HOMOs are primarily localized on the π-spacers, while the LUMOs and LUMO + 1s were primarily located on the BODIHY units. This also suggests that electronic excitations for A–D–A BODIHYs **11**–**13** have charge-transfer characteristics due to the low-energy transitions arising from contributions from the HOMO → LUMO and HOMO → LUMO + 1 excitations (Figures S24–S26). The LUMOs of **11** and **13** were very similar; however, the LUMO for **12** is delocalized through the TH bridge. The differences in optical properties within this series of compounds directly related to the identity of the bridging donor. This statement was corroborated by the fact that the HOMOs spanned the bridging donor in each compound. Furthermore, although FL-containing BODIHY **13** contains the largest π-system, it is the TH-containing BODIHY **12** that exhibited the lowest energy absorption maximum and thus the narrowest E_g_^Opt^. This observation was rationalized by comparing the angles between planes defined by the *N*-aryl substituents of the BODIHY heterocycle and the neighboring aryl ring of the bridging donor groups in the optimized structures (Figure S27). In the case of **12**, this angle was 18.8°, while in **11** (33.8°) and **13** (34.3°) the planarity, and thus the overall degree of π-conjugation, was disrupted due to the presence of two adjacent benzene rings that adopt a twisted geometry.^71,72^

### Electrochemical Properties

**Figure 5:**
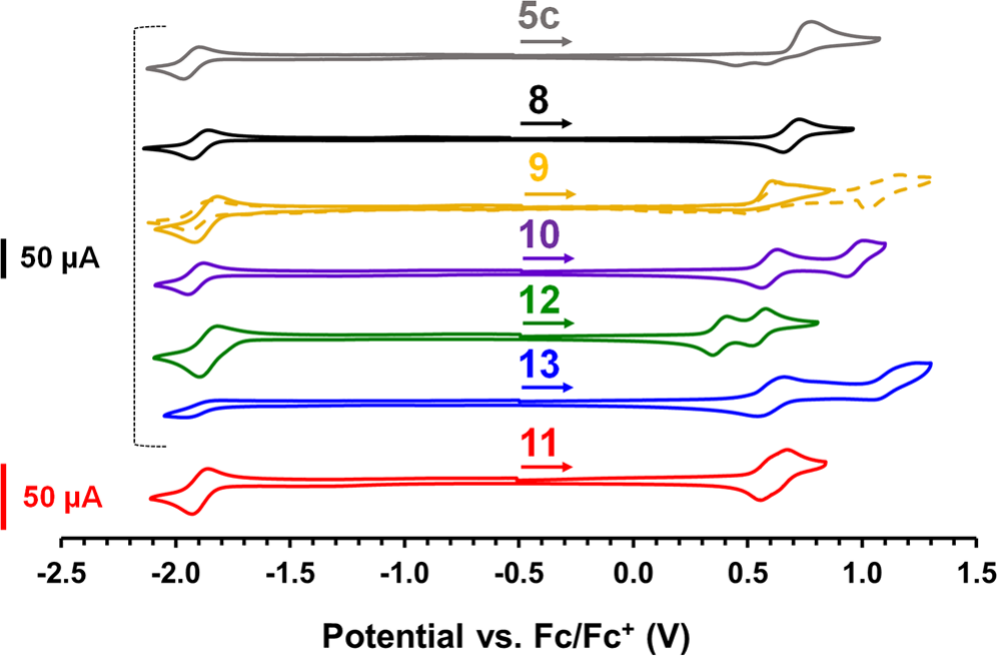
Cyclic voltammograms recorded for BODIHYs **5c** and **8–13** at a scan rate of 0.25 V s^–1^ in dry and degassed CH_2_Cl_2_ solutions containing 0.1 M [*n*Bu_4_N][PF_6_] as the supporting electrolyte and ∼1 mM analyte. The arrows denote the initial scan direction, and the dashed line is a voltammogram collected for BODIHY **9** for a wider potential window. The low current response observed for BODIHY **11** relates to its limited solubility.

**Figure 6:**
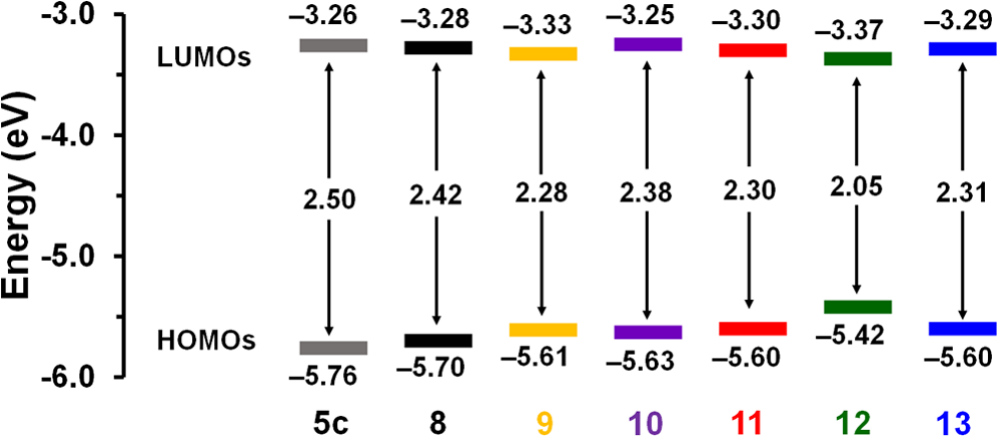
Estimated (from cyclic voltammetry (CV) data) HOMO and LUMO energies of BODIHYs **5c** and **8**–**13**.

The redox properties of BODIHYs **8**–**13** were explored using cyclic voltammetry (Figure 5 and Table 2), and all compounds exhibited reversible redox chemistry. As a point of reference, the parent BODIHY **5c** was irreversibly oxidized at 0.77 V and reduced at −1.93 V relative to the ferrocene/ferrocenium (Fc/Fc^+^) redox couple.^62^ BODIHYs **8** and **10**, which possess BE and FL substituents, respectively, each underwent reversible one-electron oxidation to produce radical cations at *E*_ox1_ = 0.69 and 0.62 V, respectively. TH-substituted BODIHY **9** exhibited irreversible oxidation waves, likely due to the reactive nature of terminal thiophene groups.^73,74^ FL-substituted BODIHY **10** exhibited a second reversible one-electron oxidation at *E*_ox2_ = 0.97 V, indicating the formation of a dication. A–D–A BODIHY **11** includes a BE spacer and exhibited two overlapping one-electron oxidations at 0.57 and 0.62 V, respectively. A–D–A BODIHYs **12** and **13**, which include TH and FL donors, respectively, each exhibited two one-electron oxidations, with the former demonstrating the lowest oxidation potential of 0.38 V, indicating that TH-containing BODIHY **12** has the highest HOMO energy in this series. The stepwise oxidations for BODIHYs **9**–**13** and highly delocalized HOMOs in **8**–**10** indicated a degree of electronic communication throughout the π-systems. With the exception of BODIHY **13**, which did not exhibit a reversible reduction, BODIHYs **8**–**12** exhibited reversible reduction waves between *E*_red1_ = −1.93 and −1.87 V, where the current response is doubled for **11** and **12**, demonstrating that each BODIHY unit simultaneously underwent a one-electron reduction. Similar to other compounds containing two BODIHY fragments,^61,63^ it can be concluded that the reductions were predominantly BODIHY based, given the similarity in reduction potentials and LUMO localization on the BODIHY fragment in compounds **8**–**13** (Figure 4). Compared to analogous BF_2_ formazanate A–D–A compounds containing TH and FL as bridging donor groups,^60^ BODIHYs **12** (*E*_ox1_ = 0.38 V, *E*_red1_ = −1.87 V) and **13** (*E*_ox1_ = 0.61 V, *E*_red1_ = −1.93 V) exhibited lower oxidation and reduction potentials. Using the onset of oxidation and reduction and the equations *E*_HOMO(CV)_ = −5.1 – *E*_onset_^ox^ and *E*_LUMO(CV)_ = −5.1 – *E*_onset_^red^, we estimated the HOMO and LUMO energies (Table 2 and Figure 6).^75^ The similar LUMO energies (−3.37 to −3.25 V) and localized LUMO densities of compounds **8**–**13** supported our conclusion that the reductions are BODIHY-based. The estimated HOMO energies ranged between −5.70 and −5.42 V and reflected the structural variation of the donor groups incorporated. The E_g_^CV^ were estimated by taking the difference between *E*_HOMO(CV)_ and *E*_LUMO(CV)_ and range from 2.05 to 2.38 eV and are generally larger than those observed for related BF_2_ formazanate A–D–A compounds.^76^ A–D–A BODIHY **12** possesses the narrowest E_g_^CV^ (2.05 eV) which was consistent with the E_g_^Opt^ of 2.02 eV and the red-shifted λ_max_ relative to other compounds described in this work. In all cases, strong agreement between the E_g_^Opt^ and E_g_^CV^ was noted, with minimal differences ranging from 0.03 to 0.19 eV (Table 3), and these band gaps were narrower than other BODIHYs reported.^66^

**Table 2:** Electrochemical Data for BODIHYs **5c** and **8–13**^a^

compound	*E*_red1_ (V)	*E*_onset_^red^ (V)	*E*_LUMO(CV)_ (V)	*E*_ox1_ (V)	*E*_ox2_ (V)	*E*_onset_^ox^ (V)	*E*_HOMO(CV)_ (V)	E_g_^CV^ (eV)
**5c**(62)	–1.93	–1.84	–3.26	0.77^b^		0.66	–5.76	2.50
**8**	–1.89	–1.82	–3.28	0.69		0.60	–5.70	2.42
**9**	–1.88	–1.77	–3.33	0.61^b^	1.16^b^	0.51	–5.61	2.28
**10**	–1.90	–1.85	–3.25	0.62	0.97	0.53	–5.63	2.38
**11**	–1.91	–1.80	–3.30	0.57^c^	0.62^c^	0.50	–5.60	2.30
**12**	–1.87	–1.73	–3.37	0.38	0.56	0.32	–5.42	2.05
**13**	–1.93^d^	–1.81	–3.29	0.61	1.22^b^	0.50	–5.60	2.31

a Cyclic voltammograms were recorded for ∼1 mM solutions of analyte in dry, degassed CH_2_Cl_2_ containing 0.1 M [*n*Bu_4_N][PF_6_] as the supporting electrolyte. The scan rate was 0.25 V s^–1^ and potentials are reported relative to the Fc/Fc^+^ redox couple.

b Irreversible process, potential at maximum anodic current reported.

c Two overlapping waves.

d Irreversible process, potential at maximum cathodic current reported.

**Table 3:** Optical Band Gaps Calculated from UV–vis Absorption Spectra and Electrochemical Band Gaps Calculated from Cyclic Voltammetry Experiments for **5c** and **8**–**13**

compound	λ_max_^onset^ (nm)	E_g_^Opt^ (eV)^a^	E_g_^CV^ (eV)	difference
**5c**	503	2.47	2.50	0.03
**8**	551	2.25	2.42	0.17
**9**	584	2.12	2.28	0.16
**10**	547	2.27	2.38	0.11
**11**	589	2.11	2.30	0.19
**12**	614	2.02	2.05	0.03
**13**	562	2.21	2.31	0.10

a E_g_^Opt^ = 1240/λ_max_^onset^.

## Conclusions

In conclusion, we report the straightforward synthesis of D–A (**8**–**10**) and A–D–A (**11**–**13**) BODIHYs with PH, TH, and FL donor units using Stille cross-coupling chemistry. All compounds offered lower-energy absorption and emission bands compared to parent BODIHY **5c** in solution and thin film, with weak emission observed in dilute solutions due to the rapid rotations and vibrations of appended rotatable substituents leading to nonradiative decay. Despite the fact that the emission intensity of BODIHYs was negatively affected by the secondary inner-filter effect, enhanced emission was observed in the solid state with Φ_em_ values up to 10%. Compounds **8**–**10**, **12**, and **13** exhibited AIE, and the properties of thin films of PMMA containing 1% BODIHY corroborated that restriction of intramolecular motion was likely the origin of aggregate- and solid-state emission. The calculated frontier molecular orbitals of D–A compounds **8**–**10** were highly delocalized while A–D–A analogues **11**–**13** possess HOMOs localized on the donor groups and LUMOs and LUMO + 1s localized on the BODIHY acceptors. Incorporating various donor groups led to the presence of multiple oxidation waves at potentials lower than those observed for related π-conjugated A–D–A materials containing BF_2_ units. Finally, the E_g_^CV^ and E_g_^Opt^ determined for BODIHYs **8**–**13** agreed well, with TH-bridged BODIHY **12** exhibiting the lowest E_g_^CV^ and E_g_^Opt^ of 2.05 and 2.02 eV, respectively. The molecular materials described herein show the narrowest band gaps reported to date for BODIHY dyes and, combined with the thin-film emission properties of these materials, this could enable their use in light-harvesting applications which has been seen for other BODIHYs.^66^ Collectively, these findings may inspire the design and development of BODIHYs to be further explored for optoelectronic applications such as organic electronics and photovoltaics.

## Experimental Section

### General Experimental Details

Unless otherwise stated, synthetic procedures were carried out under a N_2_ atmosphere using standard Schlenk techniques. Solvents were stored under a N_2_ atmosphere over 4 Å molecular sieves after they were obtained from Caledon Laboratories, dried using an Innovative Technologies Inc. solvent purification system, and collected under vacuum. Reagents were purchased from Alfa Aesar, Fisher Scientific, Oakwood Chemicals, Sigma-Aldrich, or TCI America and used as received. 2-Bromo-9,9-dihexylfluorene,^77^ 2,7-bis(trimethylstannyl)-9,9-dihexylfluorene,^78^ and BODIHY **7**(61) were prepared according to procedures reported in the literature. Stille cross-coupling reactions were conducted in an Anton Paar Monowave 50 reactor. NMR spectra were recorded on a Bruker AvanceIII HD 400 MHz spectrometer (^1^H: 399.8 MHz, ^13^C{^1^H}: 100.6 MHz, ^11^B: 128.3 MHz, ^19^F: 376.1 MHz, ^119^Sn NMR: 149.1 MHz). ^1^H NMR spectra were referenced to CHCl_3_ at 7.26 ppm or CHDCl_2_ at 5.32 ppm. ^13^C{^1^H} NMR spectra were referenced to CD_2_Cl_2_ at 54.0 ppm. ^11^B NMR spectra were reported relative to BF_3_·OEt_2_ at 0 ppm. ^19^F NMR spectra were referenced to CFCl_3_ at 0 ppm. ^119^Sn NMR spectra were referenced to SnMe_4_ at 0 ppm. Mass spectrometry studies were conducted using a Bruker MicrOTOF II instrument or a Waters Synapt HDMS instrument using electrospray ionization (positive ion mode). UV–vis absorption spectra were collected using a Cary 5000 spectrophotometer between 250 and 800 nm. Extinction coefficients were determined from the slope of a plot of absorbance *vs* concentration including data for four separate sample concentrations. A Photon Technology International (PTI) QM-4 SE spectrofluorometer was used to collect emission spectra. The absorption maxima from the respective UV–vis absorption spectrum of each compound were used as the respective excitation wavelengths. Absolute emission quantum yields were measured using a Hamamatsu C11347-11 Quantaurus Absolute Photoluminescence Quantum Yield Spectrophotometer. Fourier transform infrared (FT-IR) spectra were recorded using a PerkinElmer Spectrum Two FT-IR spectrometer equipped with an attenuated total reflectance (ATR) attachment.

### Computational Methods

The Gaussian 16 software package^79^ was used to perform molecular geometry optimizations and TDDFT calculations on a local machine or through the Graham cluster of Compute Canada using the DGDZVP2 basis set and the LC-ωhPBE^69,70^ density functional with a tuned value of the range separation parameter ω = 0.14 and the polarizable continuum model (PCM) of implicit solvation (THF). Ground-state geometries were determined by exploring various initial conformations and choosing those with the lowest energy. The lowest energy structures for all compounds were explicitly confirmed by vibrational analysis to be true minima.

### Thermal Analysis

BODIHYs were placed in a platinum pan and heated at a rate of 10 °C min^–1^ from 10 to 1000 °C under a flow of N_2_ (60 mL min^–1^) using a TA Instruments Q50 thermogravimetric analysis instrument.

### Preparation of Thin Films

The preparation of thin films of BODIHYs **8**–**13** required for absorption and emission spectroscopy was accomplished by filtering (PTFE membrane, 0.22 μm) ∼300 μL of 10 mg mL^–1^ solutions of each compound in CH_2_Cl_2_ directly onto quartz slides that were previously dipped in CH_2_Cl_2_ and air dried. The quartz slides were left at rest for 30 s before they were accelerated at a rate of 150–2000 rpm s^–1^ and spun for an additional 60 s. Thin films of PMMA doped with BODIHYs **8**–**13** were prepared similarly using a 10 mg mL^–1^ solution of 99:1 (by mass) PMMA/BODIHY.

### Aggregation Studies

Mixtures containing various ratios of degassed THF and deionized H_2_O (9.5 mL total) were combined with 0.5 mL of a 500 μM stock solution of the respective BODIHY in THF to yield 25 μM solutions with targeted H_2_O volume fractions (*f*_w_). The solutions were mixed by three inversions before their immediate analysis.

### Electrochemical Methods

Cyclic voltammograms were recorded for solutions of CH_2_Cl_2_ containing the analyte (∼1 mM) and supporting electrolyte (0.1 M [*n*Bu_4_N][PF_6_]) using a Bioanalytical Systems Inc. (BASi) Epsilon potentiostat and analyzed using BASi Epsilon software. The electrochemical cells comprised of three electrodes, including a silver wire pseudo reference electrode, a glassy carbon working electrode, and a platinum wire counter electrode. Scan rates of 50–1000 mV s^–1^ were employed, and potentials were internally referenced against the Fc/Fc^+^ redox couple (∼1 mM ferrocene as an internal standard) and corrected for internal cell resistance using the BASi Epsilon software.

### Synthetic Procedures

#### 2-Trimethylstannyl-9,9-dihexylfluorene

2-Bromo-9,9-dihexylfluorene (0.420 g, 1.02 mmol) was dissolved in THF (15 mL) before the reaction was cooled to −78 °C and stirred for 10 min. *n*BuLi (0.450 mL, 1.12 mmol) was then added dropwise causing a color change from colorless to pale yellow while the reaction was stirred for 1 h at −78 °C. In a separate flask, a solution of trimethyltinchloride (0.233 g, 1.17 mmol) in THF (2 mL) was prepared and added to the pale yellow lithium-containing solution in one portion. The reaction was then gradually warmed to room temperature and stirred for 16 h. The reaction mixture was then diluted with Et_2_O (20 mL), and the organic fraction was washed with H_2_O (15 mL), NaHCO_3_ (2 × 15 mL), H_2_O (2 × 15 mL), and brine (15 mL); dried over MgSO_4_; gravity filtered; and concentrated *in vacuo* to yield the crude product as a pale yellow solid that was used without further purification. Yield = 0.494 g, 98%. ^1^H NMR (399.8 MHz, CDCl_3_), selected assigned signals: δ 7.71–7.66 (m, 2H, aryl CH), 7.45–7.43 (m, 1H, aryl CH), 7.35–7.28 (m, 5H, aryl CH), 2.01–1.86 (m, 5H, alkyl CH), 1.16–0.94 (m, 13H, alkyl CH), 0.78–0.74 (m, 9H, alkyl CH), 0.33 (s, 9H, Sn(CH_3_)_3_). ^119^Sn NMR (149.1 MHz, CDCl_3_): δ −26.0 (s).

#### General Stille Cross-Coupling Procedures for the Synthesis of D–A BODIHYs

Compound **7** (1 equiv), trialkyltin reagent (1 equiv), Pd_2_(dba)_3_ (0.05 equiv), and P(*o*-tol)_3_ (0.1 equiv) were added to an oven-dried 10 mL glass pressure tube equipped with a rubber septum under N_2_ before dry, degassed toluene (∼5 mL) was added. The sealed tubes were then heated in an Anton Paar Monowave 50 reactor under the following conditions: (i) the temperature was ramped to 170 °C over 8 min and (ii) the temperature was held at 170 °C for 15 min with pressures reaching ∼4 bar during this time. Upon cooling to room temperature, the solutions were washed with 1 M KF_(aq)_ (3 × 10 mL), dried over MgSO_4_, gravity filtered, and concentrated *in vacuo* to afford crude reaction products that were purified by column chromatography.

#### General Stille Cross-Coupling Procedures for the Synthesis of A–D–A BODIHYs

Compound **7** (1 equiv), bis-trialkyltin reagent (0.5 equiv), Pd_2_(dba)_3_ (0.05 equiv), and P(*o*-tol)_3_ (0.1 equiv) were added to an oven-dried 10 mL glass pressure tube equipped with a rubber septum under N_2_ before dry, degassed toluene (∼5 mL) was added. The sealed tubes were then heated in an Anton Paar Monowave 50 reactor under the following conditions: (i) the temperature was ramped to 170 °C over 8 min and (ii) the temperature was held at 170 °C for 20 min with pressures reaching ∼4 bar during this time. Upon cooling to room temperature, the solutions were washed with 1 M KF_(aq)_ (3 × 10 mL), dried over MgSO_4_, gravity filtered, and concentrated *in vacuo* to afford crude mixtures that were purified by column chromatography.

##### BODIHY **8**

The compound was prepared from **7** (0.100 g, 0.250 mmol), tributylphenylstannane (0.092 g, 0.082 mL, 0.25 mmol), Pd_2_(dba)_3_ (0.011 g, 0.012 mmol), and P(*o*-tol)_3_ (0.0080 g, 0.025 mmol). The maximum pressure reached during the reaction was 3.7 bar. Column chromatography (silica gel, 3 CH_2_Cl_2_/1 hexane, *R*_f_ = 0.72) was used to purify the crude product and afforded BODIHY **8** as a red/orange solid. Yield = 0.044 g, 44%. M.p. 191–192 °C. ^1^H NMR (399.8 MHz, CD_2_Cl_2_): δ 8.78 (d, ^3^*J*_HH_ = 6 Hz, 1H, aryl CH), 8.07 (ddd, ^3^*J*_HH_ = 9 Hz, ^3^*J*_HH_ = 8 Hz, ^4^*J*_HH_ = 2 Hz, 1H, aryl CH), 7.76 (d, ^3^*J*_HH_ = 9 Hz, 2H, aryl CH), 7.73 (d, ^3^*J*_HH_ = 8 Hz, 1H, aryl CH), 7.69–7.60 (m, 7H, aryl CH), 7.56–7.41 (m, 5H, aryl CH), 7.32 (t, ^3^*J*_HH_ = 7 Hz, 1H, aryl CH). ^11^B NMR (128.3 MHz, CD_2_Cl_2_): δ 0.8 (t, ^1^*J*_BF_ = 33 Hz). ^13^C{^1^H} NMR (100.6 MHz, CD_2_Cl_2_): δ 146.2, 142.2, 141.8, 141.3, 140.5, 136.6, 136.5, 134.6, 129.8, 129.32, 129.29, 129.0, 127.8, 127.4, 127.2, 123.9, 123.3, 120.2. ^19^F NMR (376.1 MHz, CD_2_Cl_2_): δ −136.8 (q, ^1^*J*_FB_ = 31 Hz). FT-IR (ATR): 3024 (w), 2956 (w), 2922 (w), 2360 (w), 2332 (w), 1607 (m), 1486 (m), 1477 (s), 1440 (s), 1433 (s), 1356 (m), 1316 (s), 1268 (s), 1266 (s), 1226 (m), 1156 (s), 1074 (s), 1036 (s), 979 (s), 953 (m), 838 (m), 786 (s), 762 (s), 695 (s), 632 (s), 598 cm^–1^ (s). UV–vis recorded in THF: λ_max_ = 450 nm (ε = 21,500 M^–1^ cm^–1^), λ_max_ = 350 nm (ε = 4800 M^–1^ cm^–1^), λ_max_ = 289 nm (ε = 15,900 M^–1^ cm^–1^). Mass spectrometry (EI, positive mode): exact mass expected for [C_24_H_19_BF_2_N_3_]^+^, [M + H]^+^: 398.1640; found: 398.1649; difference: +2.3 ppm.

##### BODIHY **9**

The compound was prepared from **7** (0.100 g, 0.250 mmol), 2-(tributylstannyl)thiophene (0.093 g, 0.081 mL, 0.25 mmol), Pd_2_(dba)_3_ (0.011 g, 0.012 mmol), and P(*o*-tol)_3_ (0.0080 g, 0.025 mmol). The maximum pressure reached during the reaction was 4.2 bar. Column chromatography (silica gel, 3 CH_2_Cl_2_/1 hexane, *R*_f_ = 0.41) was used to purify the crude product and afforded BODIHY **9** as a red/orange solid. Yield = 0.082 g, 81%. M.p. 181–183 °C. ^1^H NMR (399.8 MHz, CD_2_Cl_2_): δ 8.79 (d, ^3^*J*_HH_ = 6 Hz, 1H, aryl CH), 8.08 (ddd, ^3^*J*_HH_ = 9 Hz, ^3^*J*_HH_ = 7 Hz, ^4^*J*_HH_ = 2 Hz, 1H, aryl CH), 7.70 (d, ^3^*J*_HH_ = 9 Hz, 3H, aryl CH), 7.67–7.58 (m, 5H, aryl CH), 7.56–7.45 (m, 3H, aryl CH), 7.31 (dd, ^3^*J*_HH_ = 4 Hz, ^3^*J*_HH_ = 1 Hz, 1H, aryl CH), 7.26 (dd, ^3^*J*_HH_ = 5 Hz, ^4^*J*_HH_ = 1 Hz, 1H, aryl CH), 7.09 (dd, ^3^*J*_HH_ = 5 Hz, ^3^*J*_HH_ = 4 Hz, 1H, aryl CH). ^11^B NMR (128.3 MHz, CD_2_Cl_2_): δ 0.7 (t, ^1^*J*_BF_ = 32 Hz). ^13^C{^1^H} NMR (100.6 MHz, CD_2_Cl_2_): δ 146.3, 144.9, 142.2, 141.8, 140.5, 136.4, 134.7, 130.1, 129.8, 129.3, 129.0, 128.6, 126.7, 124.7, 124.0, 123.4, 123.0, 120.1 (t, *J*_CF_ = 3 Hz). ^19^F NMR (376.1 MHz, CD_2_Cl_2_): δ −138.8 (q, ^1^*J*_FB_ = 29 Hz). FT-IR (ATR): 3102 (w), 3055 (w), 2956 (w), 2925 (w), 2854 (w), 2326 (w), 1618 (m), 1605 (m), 1488 (m), 1475 (s), 1440 (s), 1362 (m), 1318 (s), 1280 (s), 1233 (m), 1163 (s), 1101 (m), 1075 (s), 1038 (s), 985 (m), 952 (w), 822 (w), 784 (m), 704 (s), 632 (m), 599 cm^–1^ (s). UV–vis recorded in THF: λ_max_ = 456 nm (ε = 28,600 M^–1^ cm^–1^), λ_max_ = 306 nm (ε = 18,000 M^–1^ cm^–1^), λ_max_ = 266 nm (ε = 14,400 M^–1^ cm^–1^). Mass spectrometry (EI, positive mode): exact mass expected for [C_22_H_17_BF_2_N_3_S]^+^, [M + H]^+^: 404.1204; found: 404.1208; difference: +1.0 ppm.

##### BODIHY **10**

The compound was prepared from **7** (0.100 g, 0.250 mmol), 2-trimethylstannyl-9,9-dihexylfluorene (0.124 g, 0.250 mmol), Pd_2_(dba)_3_ (0.011 g, 0.012 mmol), and P(*o*-tol)_3_ (0.0080 g, 0.025 mmol). The maximum pressure reached during the reaction was 4.1 bar. Column chromatography (silica gel, 3 CH_2_Cl_2_/1 hexane, *R*_f_ = 0.20) was used to purify the crude product and afforded BODIHY **10** as a red/orange solid. Yield = 0.067 g, 41%. M.p. 186–188 °C. ^1^H NMR (399.8 MHz, CD_2_Cl_2_): δ 8.80 (d, ^3^*J*_HH_ = 6 Hz, 1H, aryl CH), 8.08 (ddd, ^3^*J*_HH_ = 9 Hz, ^3^*J*_HH_ = 7 Hz, ^4^*J*_HH_ = 2 Hz, 1H, aryl CH), 7.83–7.60 (m, 12H, aryl CH), 7.58–7.46 (m, 3H, aryl CH), 7.42–7.27 (m, 3H, aryl CH), 2.01 (m, 4H, alkyl CH), 1.17–0.99 (m, 12H, alkyl CH), 0.76 (t, ^3^*J*_HH_ = 7 Hz, 6H, alkyl CH), 0.71–0.59 (m, 4H, alkyl CH). ^11^B NMR (128.3 MHz, CD_2_Cl_2_): δ 0.8 (t, ^1^*J*_BF_ = 31 Hz). ^13^C{^1^H} NMR (100.6 MHz, CD_2_Cl_2_): δ 152.0, 151.6, 146.1, 142.2, 141.9, 141.4, 140.6, 140.5, 140.2, 137.2, 136.5, 134.5, 129.8, 129.3, 129.0, 127.8, 127.5, 127.3, 126.0, 123.9, 123.5, 123.4, 121.7, 120.4, 120.2, 55.7, 40.9, 32.1, 30.3, 24.4, 23.1, 14.3. ^19^F NMR (376.1 MHz, CD_2_Cl_2_): δ −138.7 (m). FT-IR (ATR): 3055 (w), 3023 (w), 2953 (m), 2929 (s), 2852 (m), 2356 (w), 2326 (w), 1733 (w), 1619 (m), 1605 (w), 1509 (m), 1477 (s), 1426 (m), 1362 (m), 1285 (s), 1263 (s), 1233 (w), 1162 (s), 1127 (m), 1093 (m), 1068 (s), 1042 (s), 984 (m), 887 (w), 822 (m), 778 (s), 735 (s), 702 (s), 636 (m), 599 (m), 553 cm^–1^ (m). UV–vis recorded in THF: λ_max_ = 457 nm (ε = 12,500 M^–1^ cm^–1^), λ_max_ = 321 nm (ε = 12,500 M^–1^ cm^–1^), λ_max_ = 276 nm (ε = 8000 M^–1^ cm^–1^). Mass spectrometry (EI, positive mode): exact mass expected for [C_43_H_47_BF_2_N_3_]^+^, [M + H]^+^: 654.3831; found: 654.3818; difference: −2.0 ppm.

##### BODIHY **11**

The compound was prepared from **7** (0.100 g, 0.250 mmol), 1,4-bis(tributylstannyl)benzene (0.082 g, 0.072 mL, 0.13 mmol), Pd_2_(dba)_3_ (0.011 g, 0.012 mmol), and P(*o*-tol)_3_ (0.0080 g, 0.025 mmol). The maximum pressure reached was 3.8 bar. Column chromatography (silica gel, 3 CH_2_Cl_2_/1 hexane, *R*_f_ = 0.22) was used to purify the crude product and afforded BODIHY **11** as a red/orange solid. M.p. > 250 °C. ^1^H NMR (399.8 MHz, CD_2_Cl_2_): δ 8.81 (d, ^3^*J*_HH_ = 6 Hz, 2H, aryl CH), 8.09 (ddd,^3^*J*_HH_ = 9 Hz, ^3^*J*_HH_ = 7 Hz, ^4^*J*_HH_ = 2 Hz, 2H, aryl CH), 7.82–7.62 (m, 20H, aryl CH), 7.57–7.45 (m, 6H, aryl CH). ^11^B NMR (128.3 MHz, CD_2_Cl_2_): δ 0.8 (m). ^19^F NMR (376.1 MHz, CD_2_Cl_2_): δ −138.7 (m). Due to the limited solubility of **11**, ^13^C{^1^H} NMR data was not acquired despite several attempts. FT-IR (ATR): 3024 (w), 2919 (m), 2848 (w), 2356 (w), 2329 (w), 1617 (m), 1474 (s), 1424 (m), 1430 (s), 1362 (m), 1321 (s), 1277 (s), 1268 (s), 1164 (s), 1099 (m), 1073 (s), 1053 (s), 1031 (s), 985 (s), 816 (s), 779 (s), 775 (s), 702 (s), 630 cm^–1^ (s). UV–vis recorded in THF: λ_max_ = 468 nm (ε = 42,800 M^–1^ cm^–1^), λ_max_ = 310 nm (ε = 21,900 M^–1^ cm^–1^), λ_max_ = 258 nm (ε = 15,100 M^–1^ cm^–1^). Mass spectrometry (EI, positive mode): exact mass expected for [C_42_H_31_B_2_F_4_N_6_]^+^, [M + H]^+^: 717.2732; found: 717.2718; difference: −2.0 ppm.

##### BODIHY **12**

The compound was prepared from **7** (0.100 g, 0.250 mmol), 2,5-bis(tributylstannyl)thiophene (0.083 g, 0.070 mL, 0.13 mmol), Pd_2_(dba)_3_ (0.011 g, 0.012 mmol), and P(*o*-tol)_3_ (0.0080 g, 0.025 mmol). The maximum pressure reached during the reaction was 4.3 bar. Column chromatography (silica gel, 3 CH_2_Cl_2_/1 hexane, *R*_f_ = 0.21) was used to purify the crude product and afforded BODIHY **12** as a red/orange solid. Yield = 0.054 g, 30%. M.p. > 250 °C. ^1^H NMR (399.8 MHz, CD_2_Cl_2_): δ 8.80 (d, ^3^*J*_HH_ = 6 Hz, 2H, aryl CH), 8.08 (ddd, ^3^*J*_HH_ = 9 Hz, ^3^*J*_HH_ = 7 Hz, ^4^*J*_HH_ = 2 Hz, 2H, aryl CH), 7.74 (d, ^3^*J*_HH_ = 8 Hz, 6H, aryl CH), 7.69–7.62 (m, 10H, aryl CH), 7.56–7.46 (m, 6H, aryl CH), 7.30 (s, 2H, aryl CH). ^11^B NMR (128.3 MHz, CD_2_Cl_2_): δ 0.7 (m). ^13^C{^1^H} NMR (100.6 MHz, CD_2_Cl_2_): δ 143.5, 142.2, 141.9, 140.5, 136.4, 130.1, 129.8, 129.3, 129.0, 126.3, 124.0, 123.9, 123.4, 120.1. ^19^F NMR (376.1 MHz, CD_2_Cl_2_): δ −138.8 (m). FT-IR (ATR): 3058 (w), 3029 (w), 2354 (w), 2322 (w), 1615 (w), 1602 (w), 1563 (w), 1470 (s), 1430 (s), 1418 (s), 1359 (m), 1318 (s), 1273 (s), 1229 (m), 1149 (s), 1148 (s), 1105 (s), 1075 (s), 1036 (s), 972 (s), 945 (m), 840 (m), 803 (s), 776 (s), 750 (s), 704 (s), 630 (s), 598 cm^–1^ (s). UV–vis recorded in THF: λ_max_ = 487 nm (ε = 49,900 M^–1^ cm^–1^), λ_max_ = 354 nm (ε = 18,000 M^–1^ cm^–1^), λ_max_ = 299 nm (ε = 12,800 M^–1^ cm^–1^), λ_max_ = 264 nm (ε = 18,800 M^–1^ cm^–1^). Mass spectrometry (EI, positive mode): exact mass expected for [C_40_H_29_B_2_F_4_N_6_S]^+^, [M + H]^+^: 723.2297; found: 723.2268; difference: −4.0 ppm.

##### BODIHY **13**

The compound was prepared from **7** (0.100 g, 0.250 mmol), 2,7-bis(trimethylstannyl)-9,9-dihexylfluorene (0.083 g, 0.13 mmol), Pd_2_(dba)_3_ (0.011 g, 0.012 mmol), and P(*o*-tol)_3_ (0.0080 g, 0.025 mmol). The maximum pressure reached during the reaction was 3.2 bar. Column chromatography (silica gel, 3 CH_2_Cl_2_/1 hexane, *R*_f_ = 0.72) was used to purify the crude product and afforded BODIHY **13** as a red/orange solid. Yield = 0.090 g, 37%. M.p. 144–146 °C. ^1^H NMR (399.8 MHz, CD_2_Cl_2_): δ 8.81 (d, ^3^*J*_HH_ = 6 Hz, 2H, aryl CH), 8.08 (ddd, ^3^*J*_HH_ = 9 Hz, ^3^*J*_HH_ = 7 Hz, ^3^*J*_HH_ = 2 Hz, 2H, aryl CH), 7.81 (dd, ^3^*J*_HH_ = 8 Hz, ^3^*J*_HH_ = 6 Hz, 6H, aryl CH), 7.77–7.61 (m, 16H, aryl CH), 7.58–7.46 (m, 6H, aryl CH), 2.15–2.05 (m, 4H, alkyl CH), 1.18–1.02 (m, 12H, alkyl CH), 0.78–0.71 (m, 10H, alkyl CH). ^11^B NMR (128.3 MHz, CD_2_Cl_2_): δ 0.8 (m). ^13^C{^1^H} NMR (100.6 MHz, CD_2_Cl_2_): δ 152.3, 146.1, 142.2, 141.9, 140.5, 140.4, 140.1, 137.2, 136.5, 134.5, 129.8, 129.3, 129.0, 127.8, 126.1, 123.9, 123.4, 121.7, 120.4, 120.2, 55.8, 41.0, 32.1, 30.3, 24.5, 23.1, 14.3. ^19^F NMR (376.1 MHz, CD_2_Cl_2_): δ −138.7 (m). FT-IR (ATR): 3069 (w), 3028 (w), 2950 (m), 2928 (m), 2855 (m), 2328 (w), 1617 (m), 1606 (m), 1569 (m), 1488 (m), 1478 (s), 1464 (s), 1434 (s), 1362 (m), 1314 (m), 1278 (s), 1233 (m), 1165 (s), 1105 (m), 1074 (m), 1044 (m), 986 (m), 952 (w), 819 (m), 785 (m), 703 (m), 630 (m), 599 cm^–1^ (m). UV–vis recorded in THF: λ_max_ = 466 nm (ε = 58,900 M^–1^ cm^–1^), λ_max_ = 344 nm (ε = 34,300 M^–1^ cm^–1^), λ_max_ = 306 nm (ε = 11,400 M^–1^ cm^–1^), λ_max_ = 271 nm (ε = 15,200 M^–1^ cm^–1^). Mass spectrometry (EI, positive mode): exact mass expected for [C_61_H_59_B_2_F_4_N_6_]^+^, [M + H]^+^: 973.4923; found: 973.4927; difference: +0.4 ppm.

## Supporting Information Available

Copies of NMR spectra, photophysical data, and details of the theoretical calculations (PDF)
