# Supplementary material for: Tuning the Properties of Donor–Acceptor and Acceptor–Donor–Acceptor Boron Difluoride Hydrazones via Extended π-Conjugation
Source: ACS Omega. 2022 Aug 26;7(36):32727–39. doi: 10.1021/acsomega.2c04401 (PMC9476501; doi:10.1021/acsomega.2c04401)
Supplement: Supplementary file 1 — ao2c04401_si_001.pdf [file ao2c04401_si_001.pdf]

Supporting Information

Tuning the Properties of Donor-Acceptor and Acceptor-Donor-Acceptor Boron Difluoride Hydrazones via Extended π-Conjugation

Daniela Cappello, Francis L. Buguis, and Joe B. Gilroy

Department of Chemistry and the Centre for Advanced Materials and Biomaterials Research (CAMBR), The University of Western Ontario, London, Ontario, N6A 5B7, Canada.

Table of Contents

NMR SPECTRA.. S2
ADDITIONAL CHARACTERIZATION DATA S10
COMPUTATIONAL DETAILS S15

NMR SPECTRA

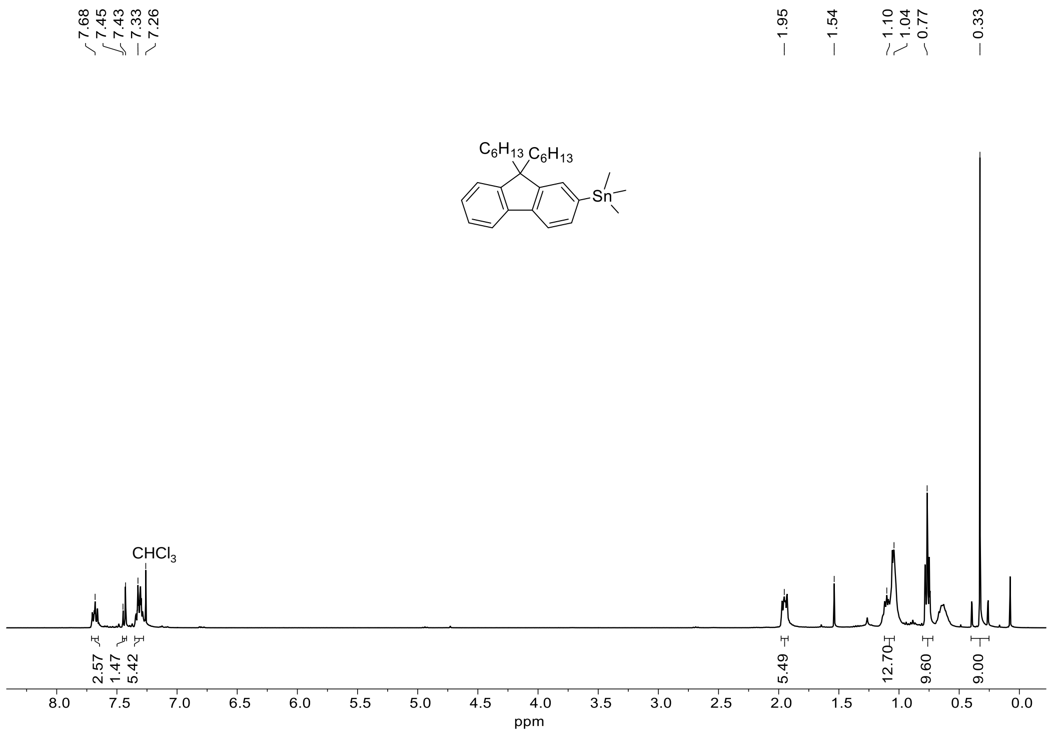

Figure S1. ^{1}H NMR spectrum of 2-trimethylstannyl-9,9-dihexylfluorene in $CDCl_3$.

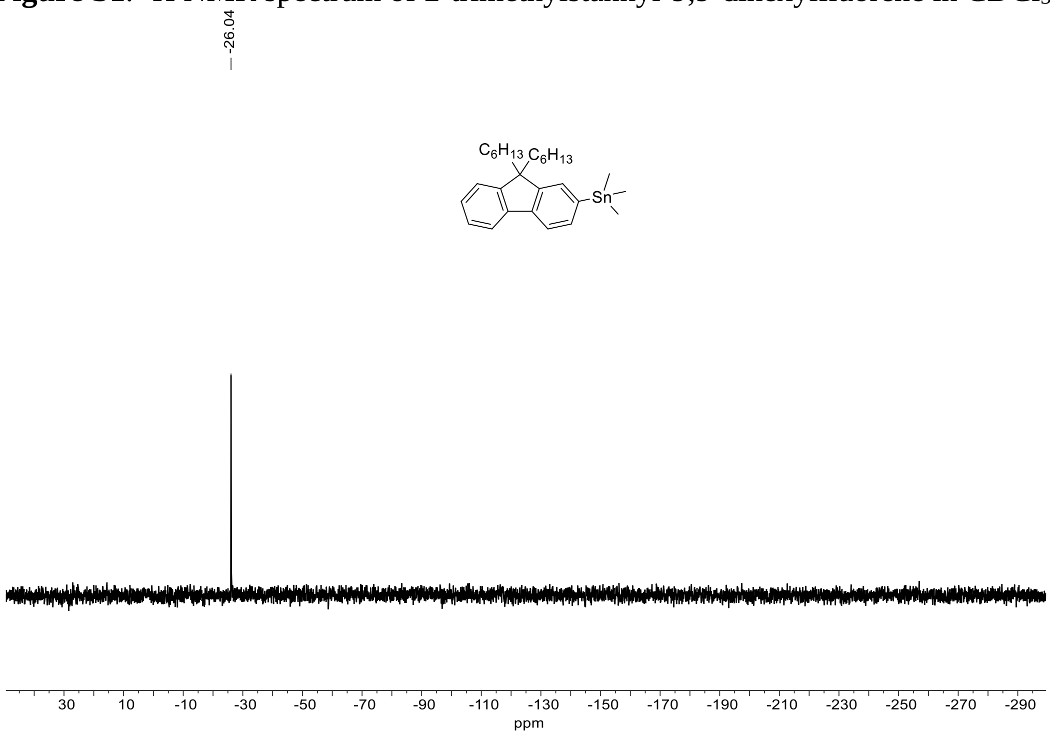

Figure S2. ^{119}Sn NMR spectrum of 2-trimethylstannyl-9,9-dihexylfluorene in $CDCl_3$.

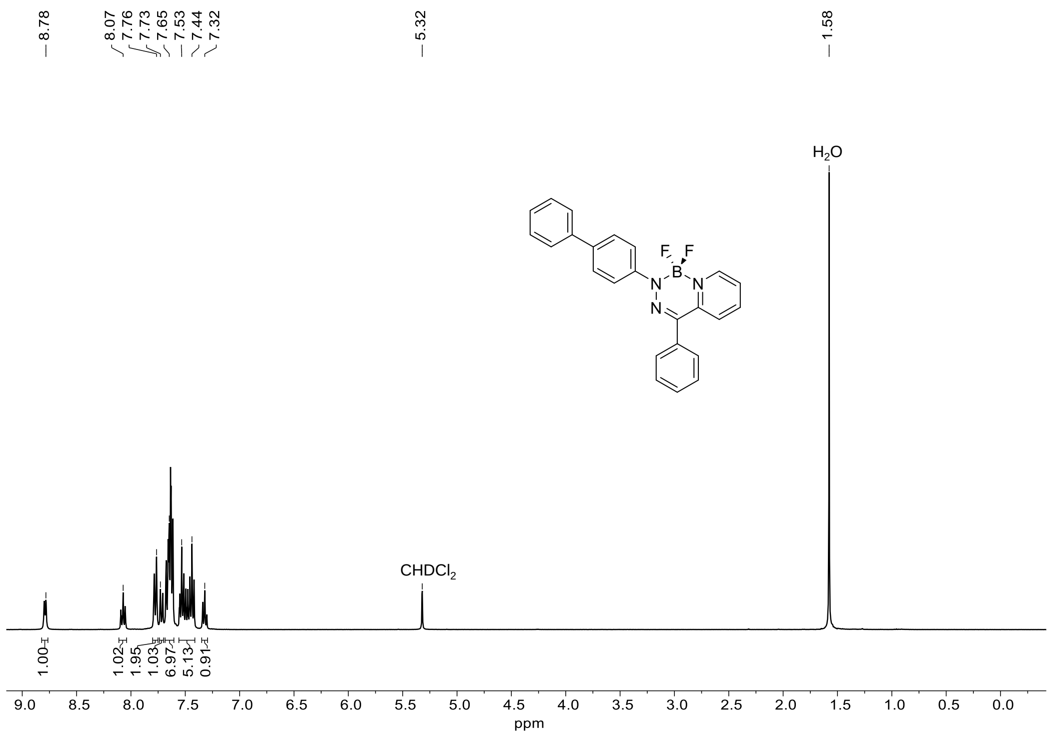

Figure S3. ^{1}H NMR spectrum of BODIHY **8** recorded in CD_2Cl_2.

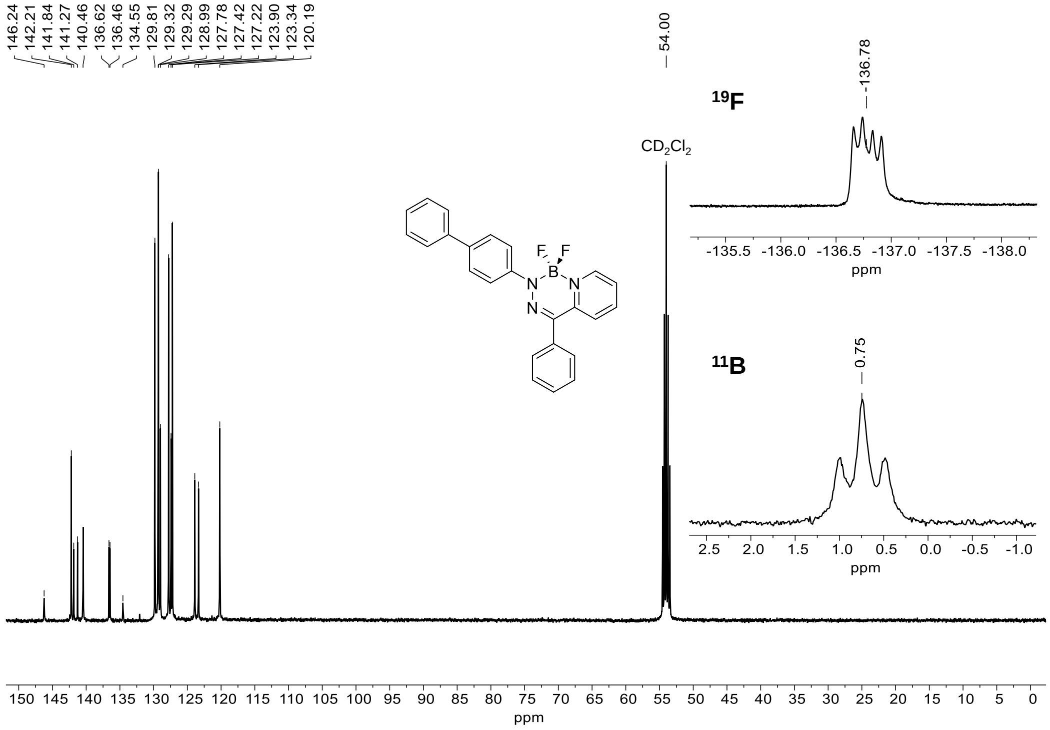

Figure S4. $^{13}C\{^{1}H\}$, ^{19}F, and ^{11}B NMR spectrum of BODIHY **8** recorded in CD_2Cl_2.

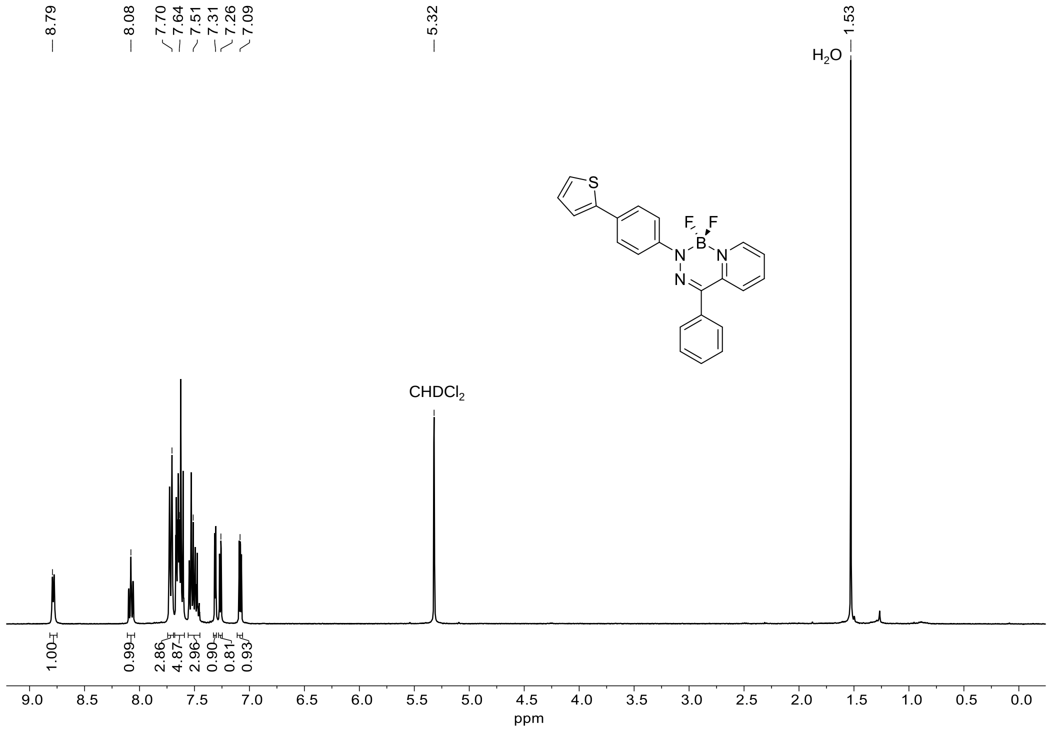

Figure S5. ^{1}H NMR spectrum of BODIHY **9** recorded in CD_2Cl_2.

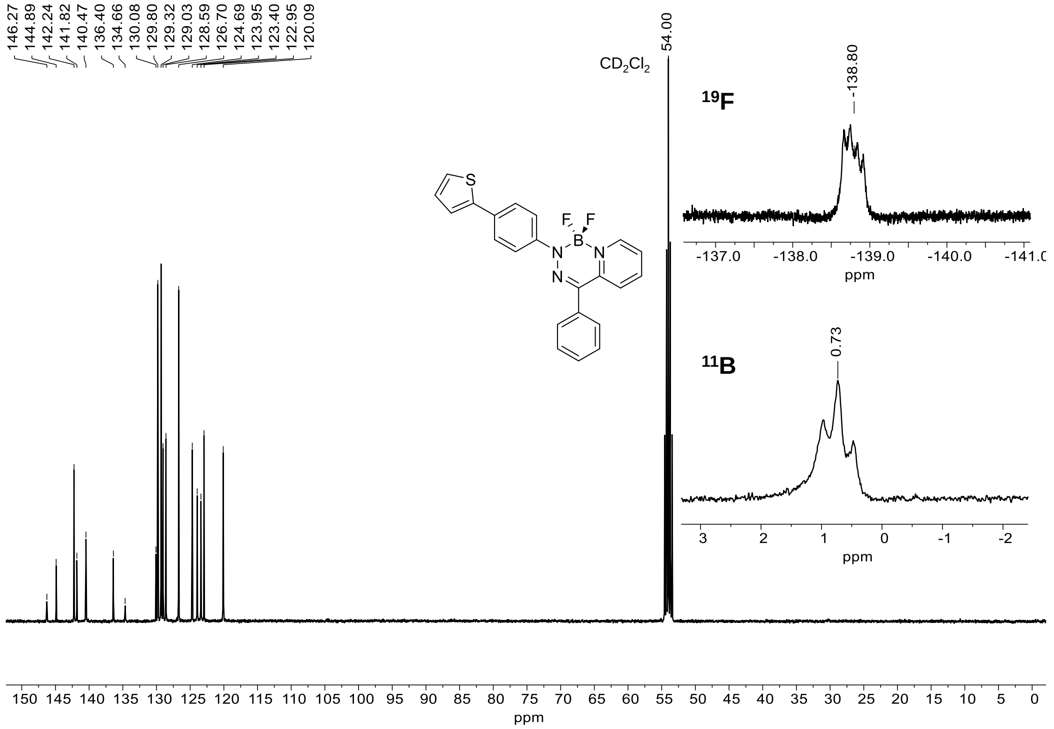

Figure S6. $^{13}C\{^{1}H\}$, ^{19}F, and ^{11}B NMR spectrum of BODIHY **9** recorded in CD_2Cl_2.

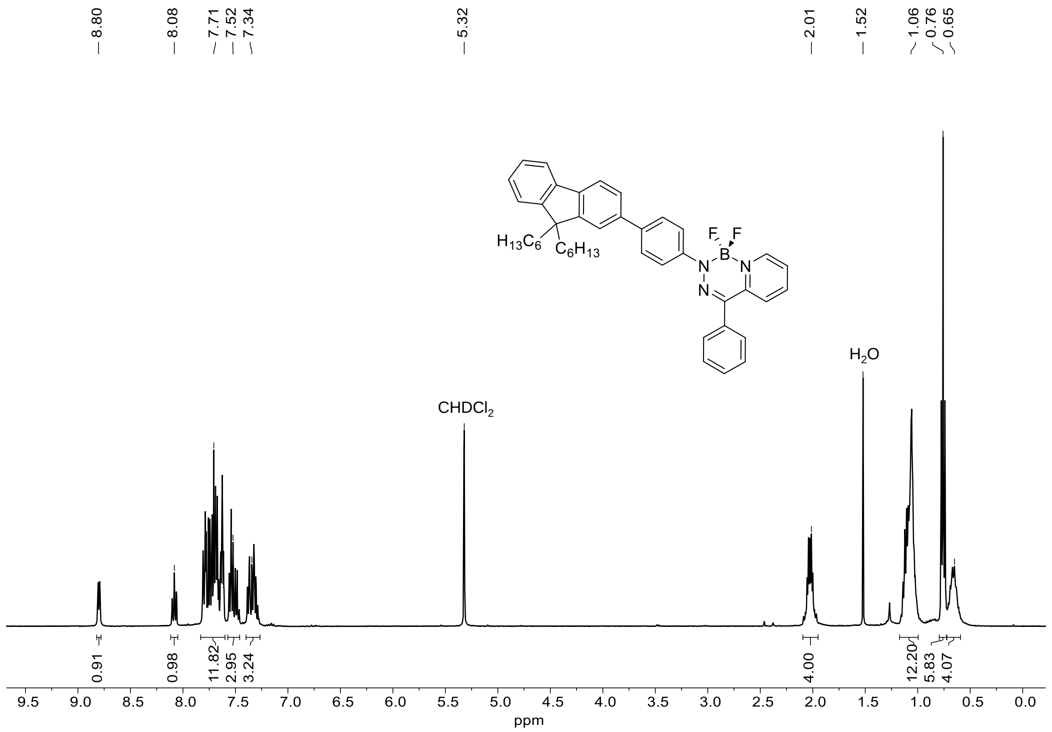

Figure S7. ^{1}H NMR spectrum of BODIHY **10** recorded in CD_2Cl_2.

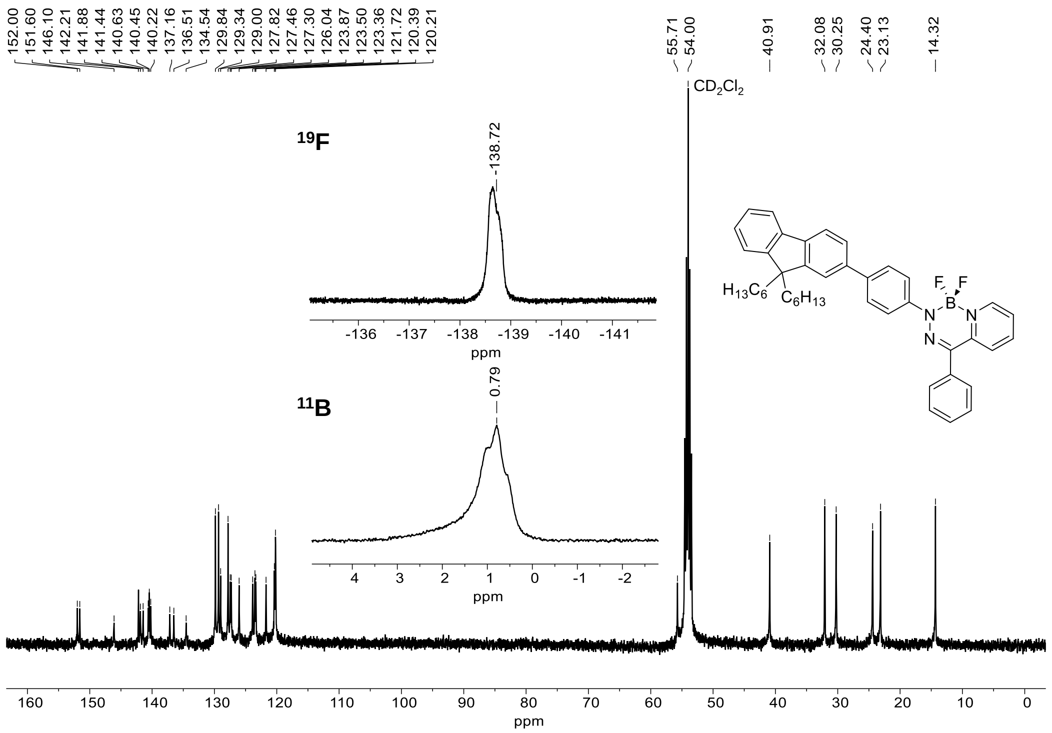

Figure S8. $^{13}C\{^{1}H\}$, ^{19}F, and ^{11}B NMR spectrum of BODIHY **10** recorded in CD_2Cl_2.

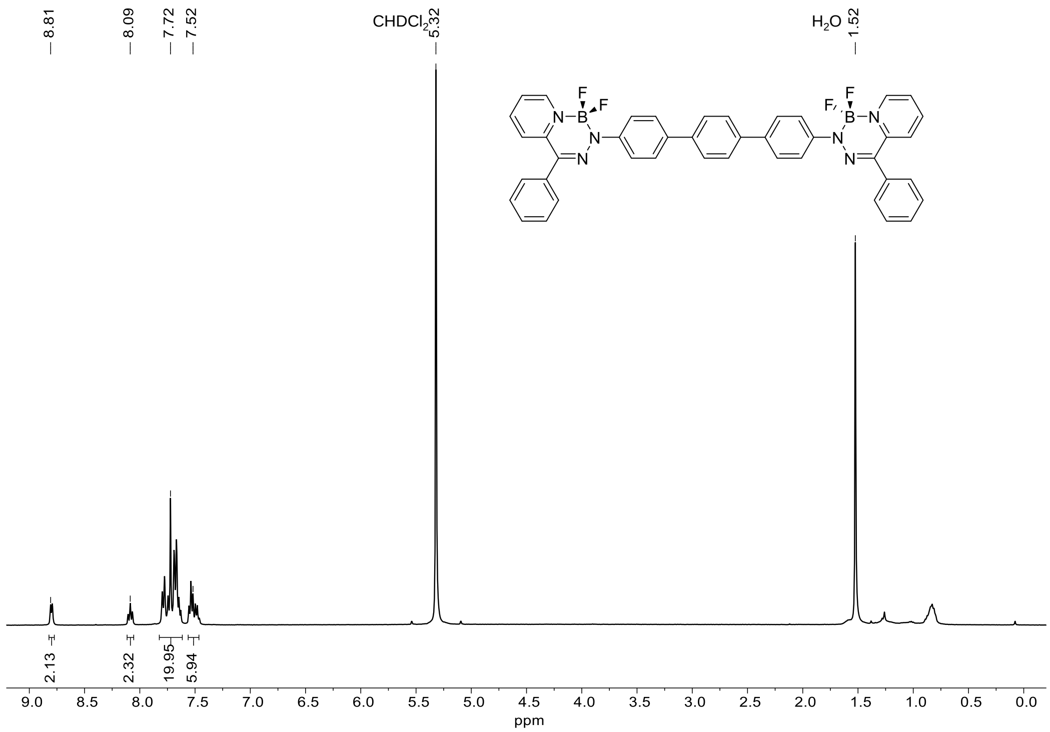

Figure S9. ^{1}H NMR spectrum of BODIHY **11** recorded in CD_2Cl_2.

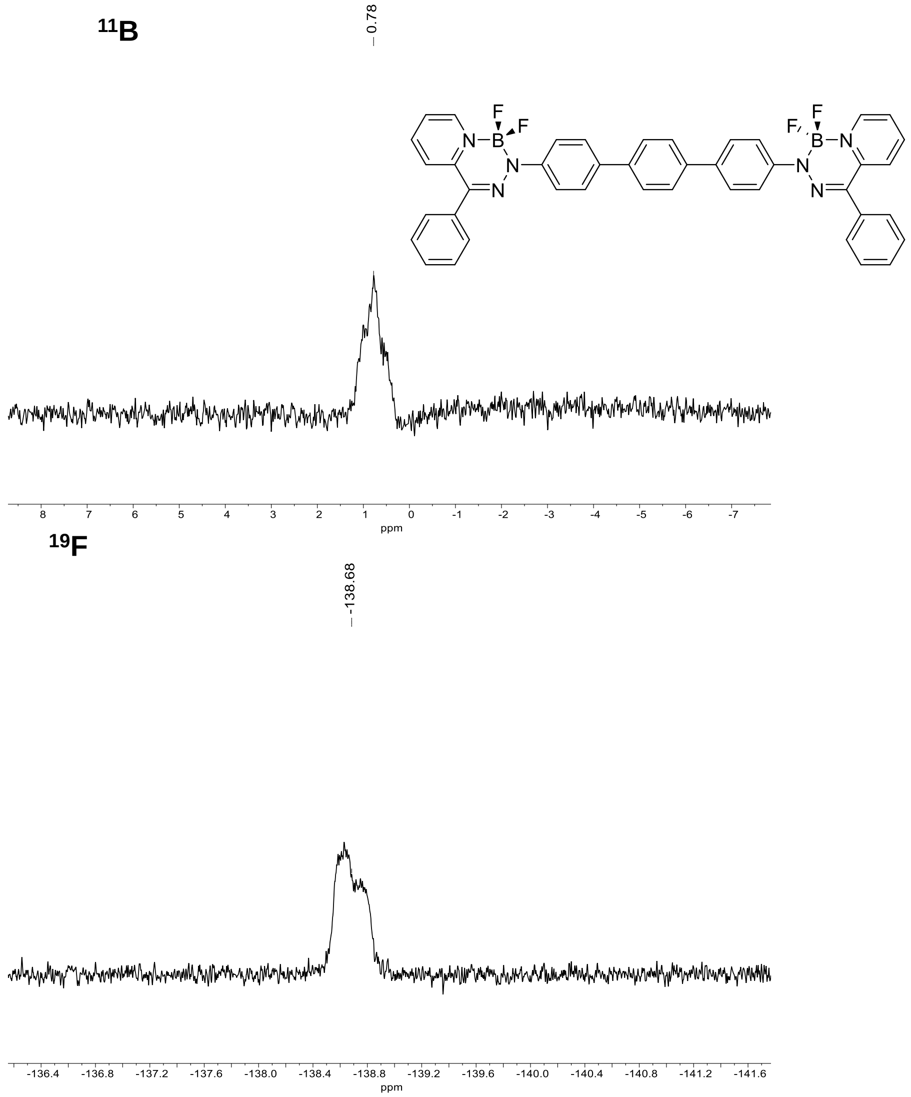

Figure S10. ^{11}B and ^{19}F NMR spectrum of BODIHY **11** recorded in CD_2Cl_2.

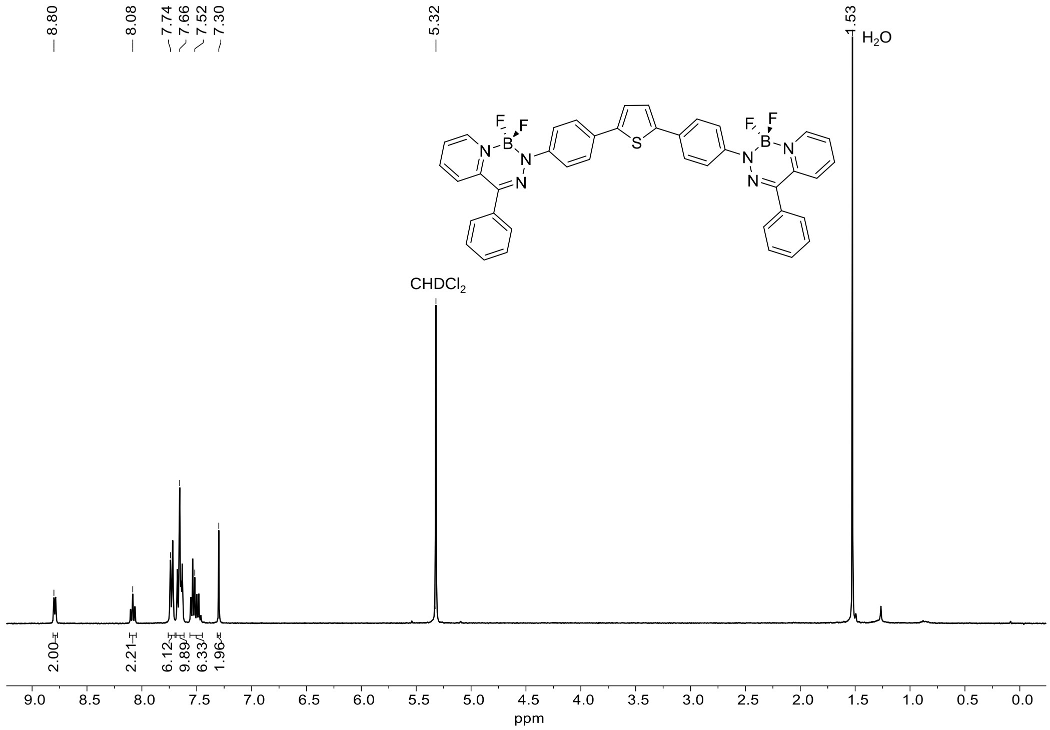

Figure S11. ^{1}H NMR spectrum of BODIHY **12** recorded in CD_2Cl_2.

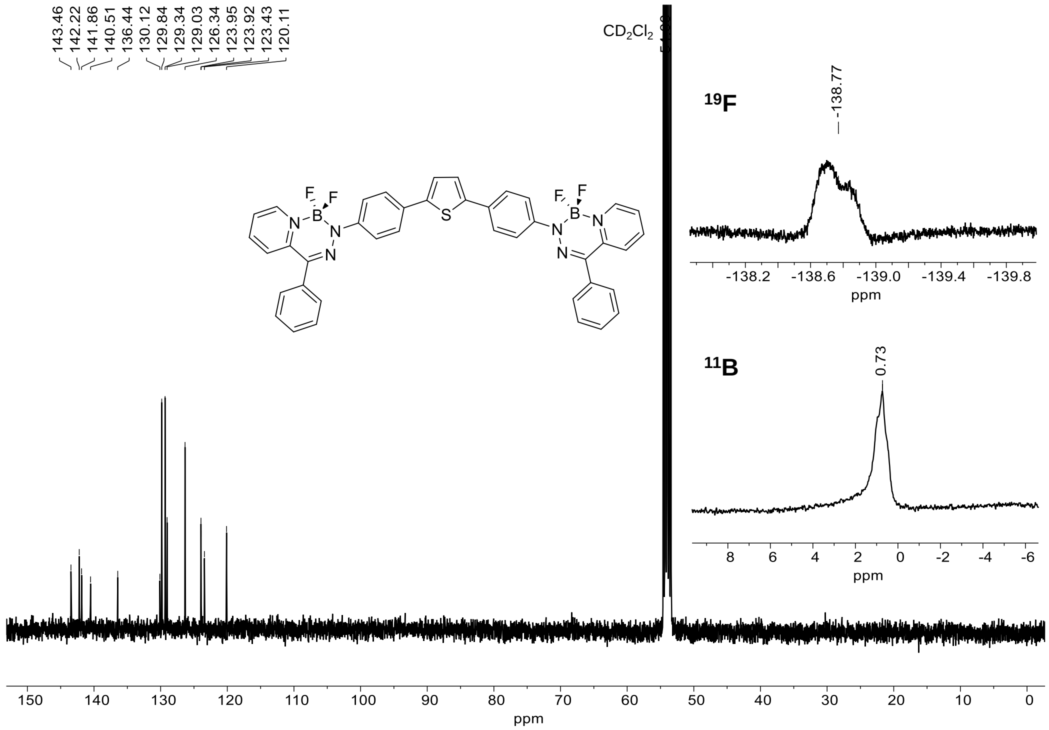

Figure S12. $^{13}C\{^1H\}$, ^{19}F, and ^{11}B NMR spectrum of BODIHY **12** recorded in CD_2Cl_2.

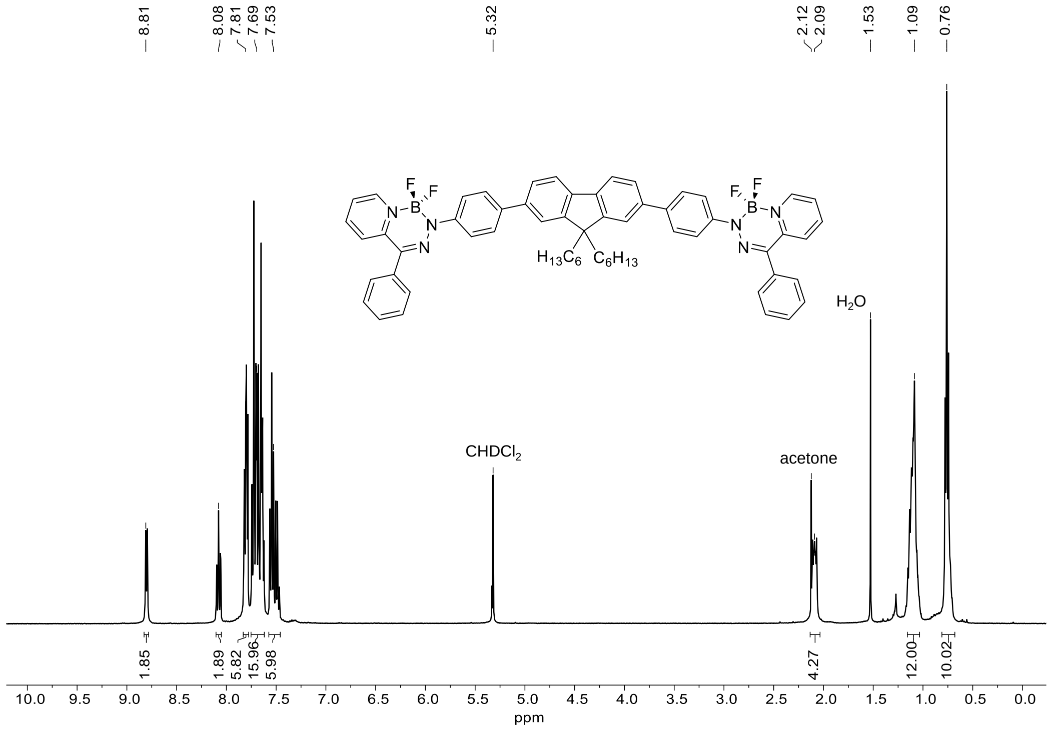

Figure S13. ^{1}H NMR spectrum of BODIHY **13** recorded in CD_2Cl_2.

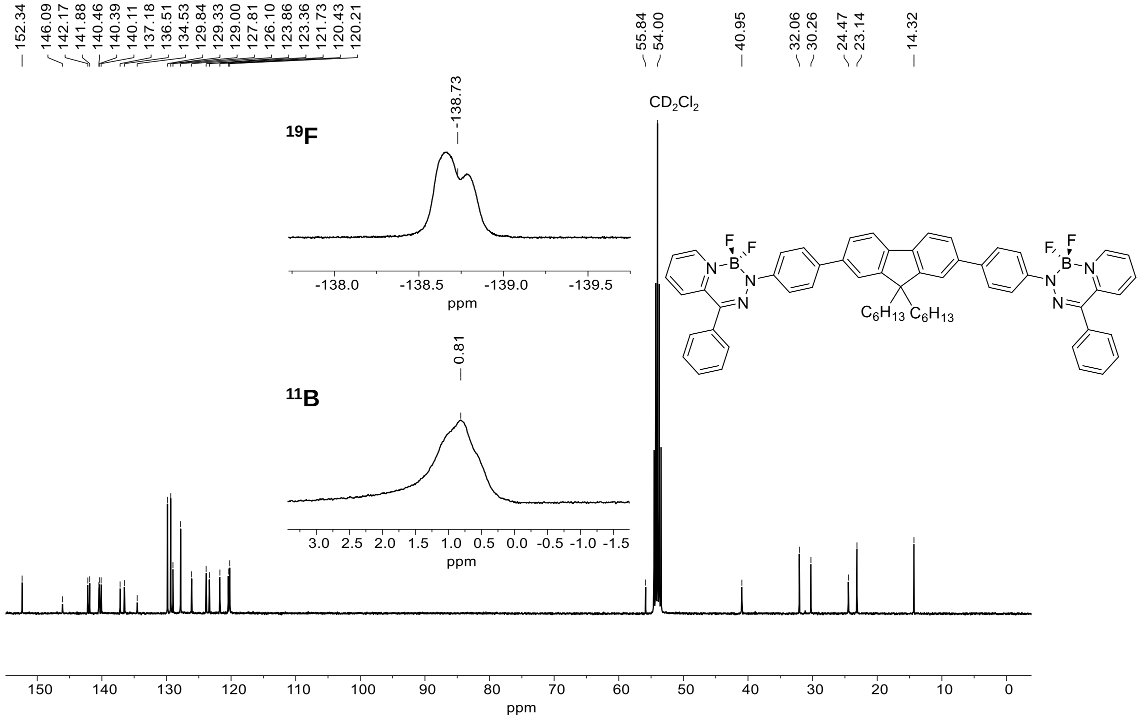

Figure S14. $^{13}C\{^{1}H\}$, ^{19}F, and ^{11}B NMR spectrum of BODIHY **13** recorded in CD_2Cl_2.

ADDITIONAL CHARACTERIZATION DATA

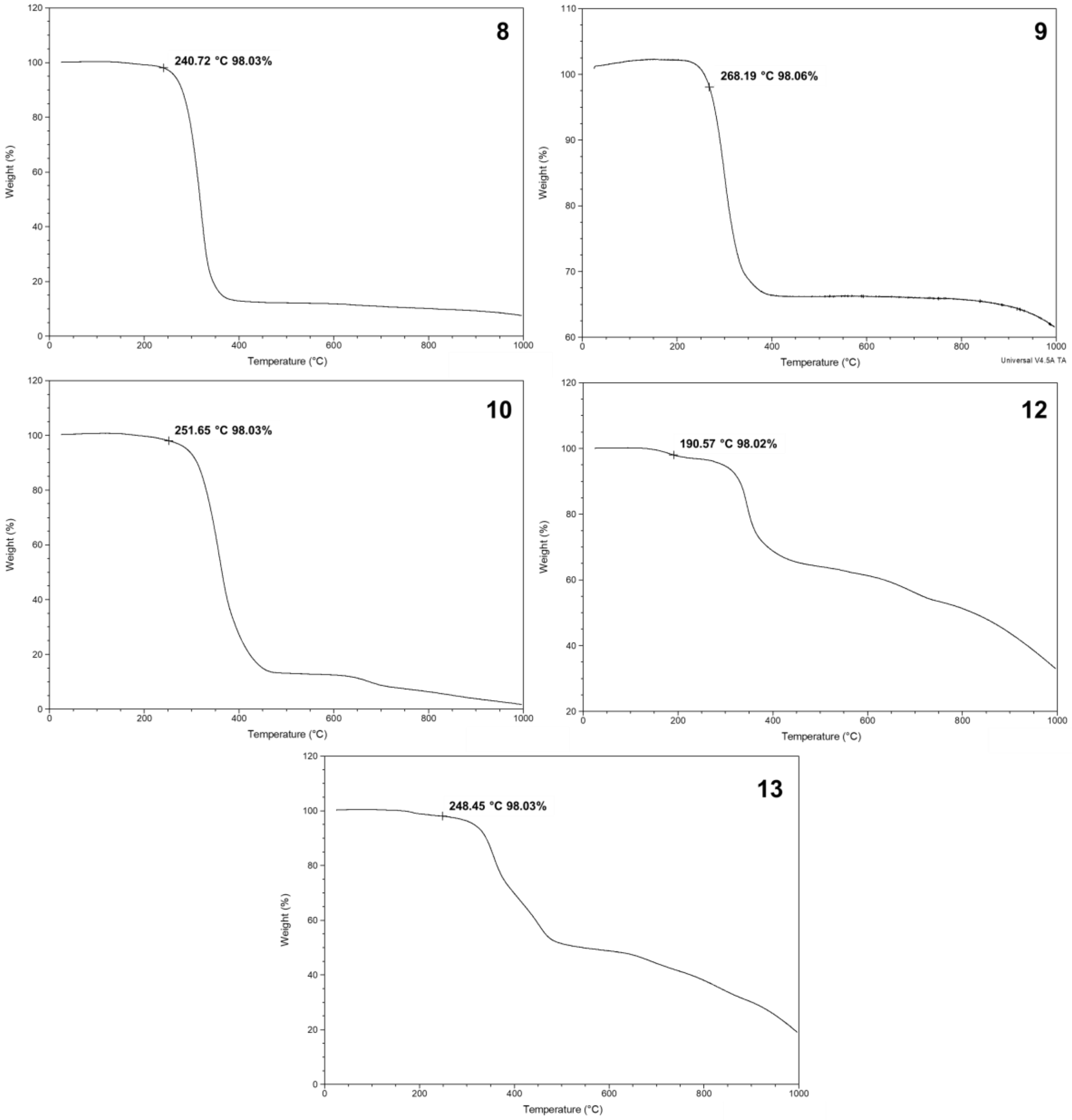

Figure S15. TGA data collected for BODIHYs **8**–**10**, **12**, and **13** under an atmosphere of N_2. The onset of thermal decomposition was taken as the temperature at 2% mass loss.

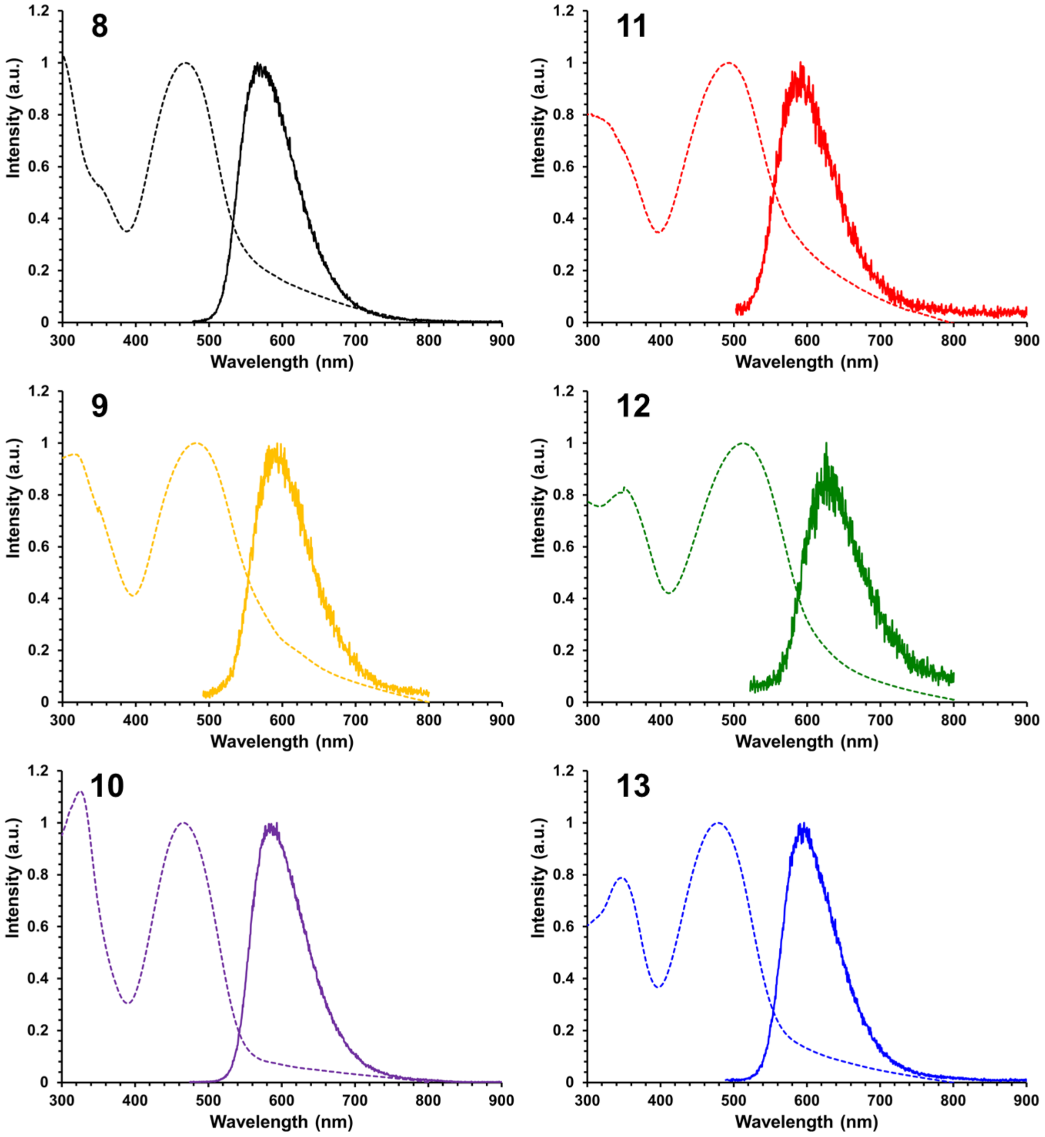

Figure S16. Absorption spectra (dashed) and emission spectra (solid) collected for thin films of compounds **8–13**.

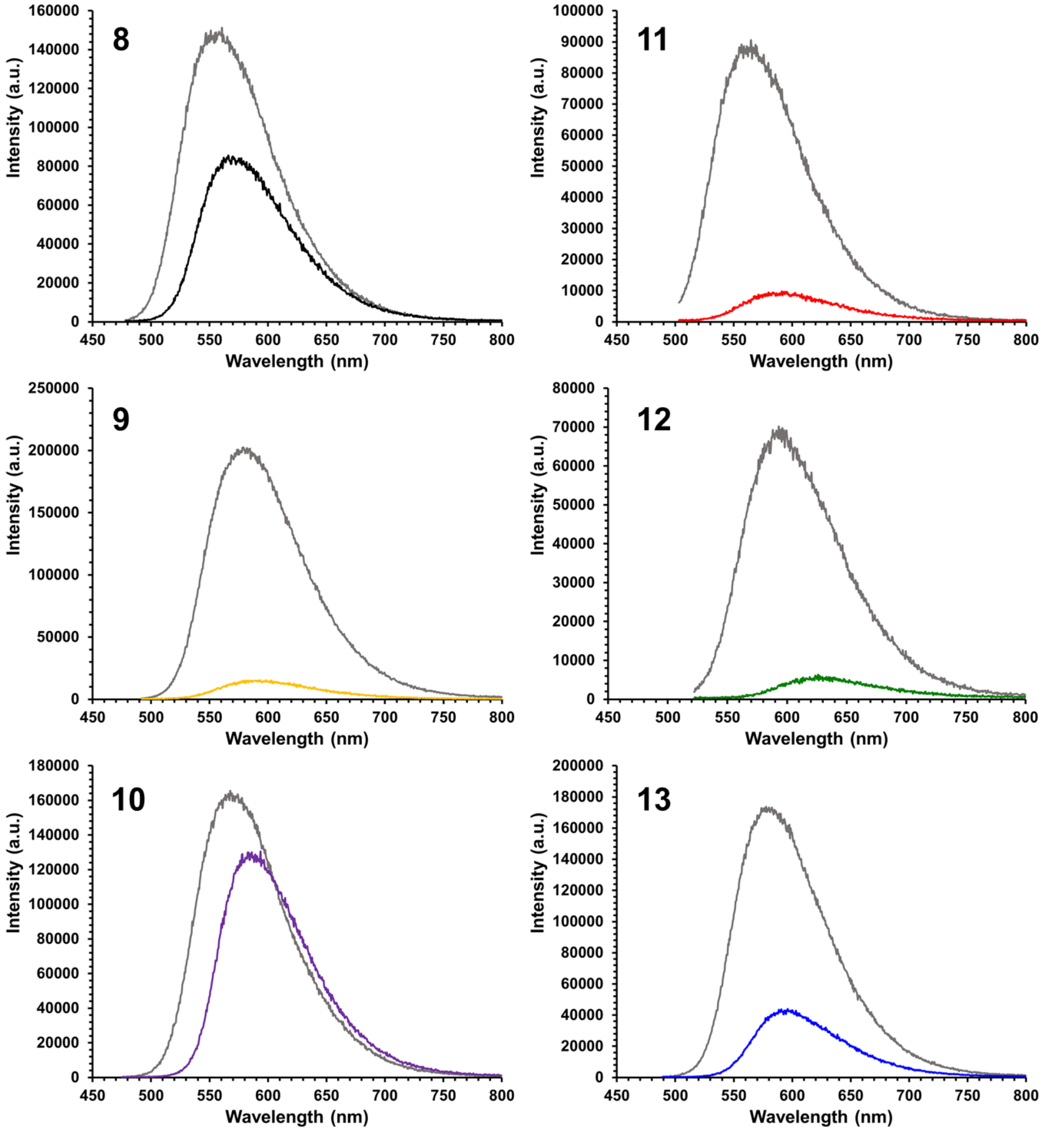

Figure S17. Emission spectra collected for compounds **8–13** with wt% PMMA:BODIHY 99:1 (grey) plotted with thin film emission spectra collected for pure compounds **8–13** (coloured).

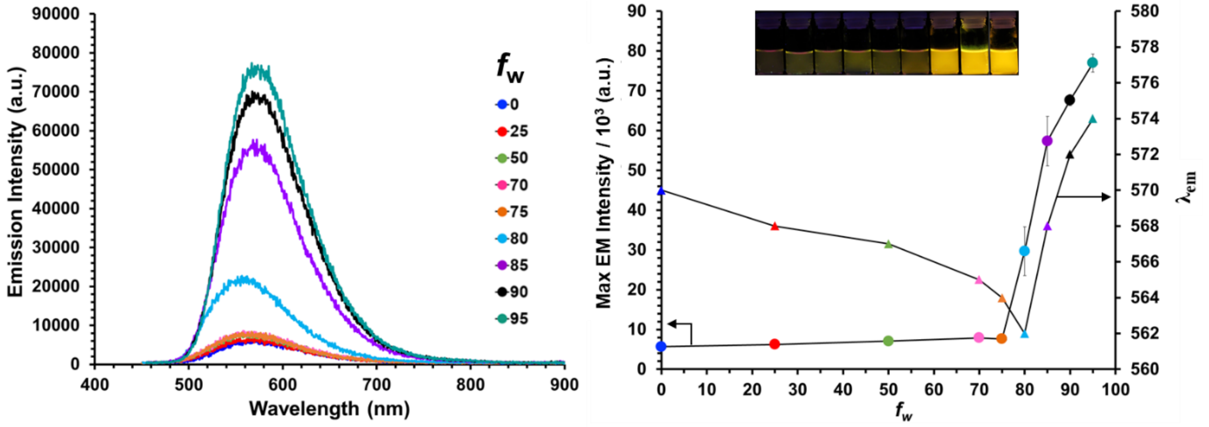

Figure S18. Left: Emission spectra for solutions of **8** at various f_w in THF. Right: Maximum emission intensities (circles) and λ_{em} (triangles) for **8** plotted as a function of f_w in THF. Concentrations were 25 μM of the analyte. Each data point represents the average from experiments conducted in triplicate.

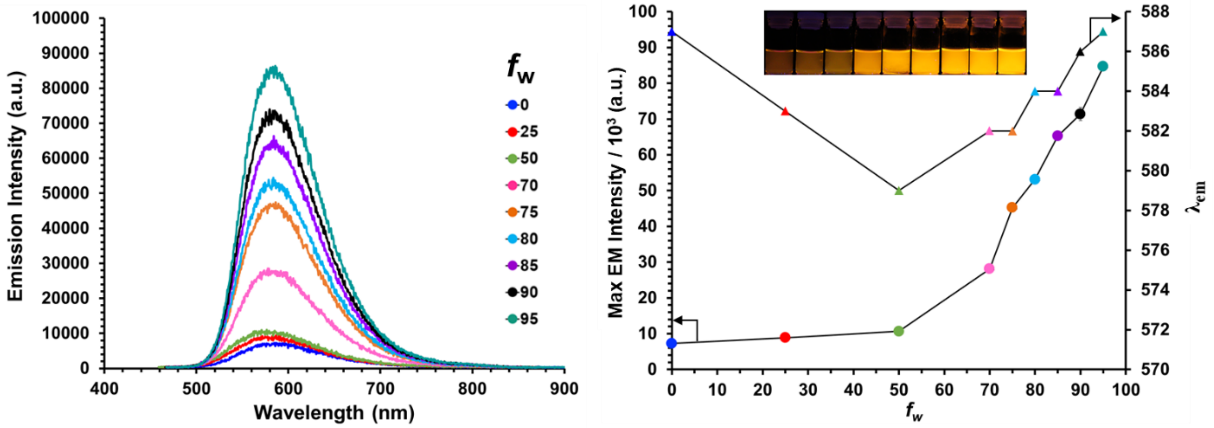

Figure S19. Left: Emission spectra for solutions of **10** at various f_w in THF. Right: Maximum emission intensities (circles) and λ_{em} (triangles) for **10** plotted as a function of f_w in THF. Concentrations were 25 μM of the analyte. Each data point represents the average from experiments conducted in triplicate.

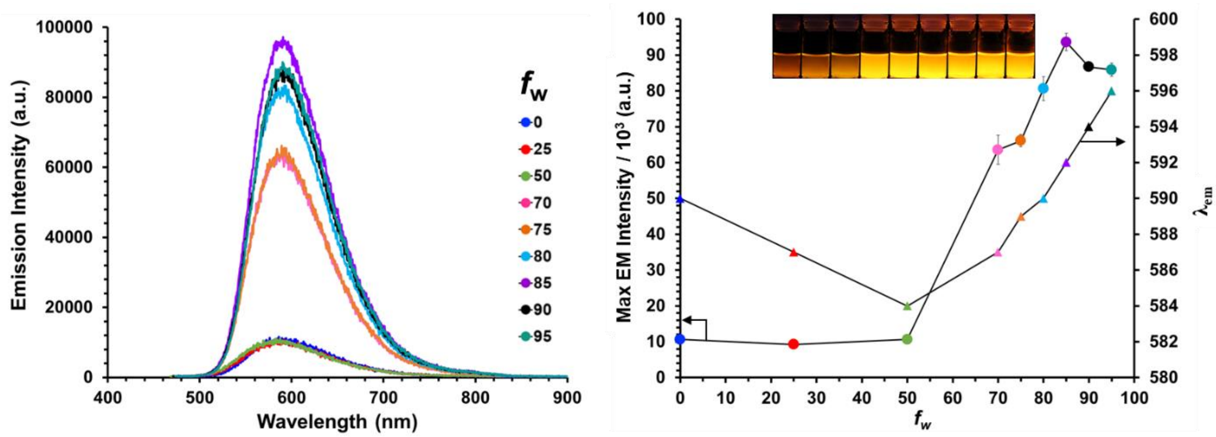

Figure S20. Left: Emission spectra for solutions of **13** at various f_w in THF. Right: Maximum emission intensities (circles) and λ_{em} (triangles) for **13** plotted as a function of f_w in THF. Concentrations were 25 μM of the analyte. Each data point represents the average from experiments conducted in triplicate.

COMPUTATIONAL DETAILS

Table S1. Experimental and calculated UV-Vis absorption band maxima of **8**–**13** in THF solution and calculated using adiabatic linear-response time-dependent LC-ωhPBE(ω=0.14)/DGDZVP2 method and the polarizable continuum model of implicit solvation.

	**Experiment**		**Theory**		
	λ_{max}^{exp} (nm)	**ε (M^{-1} cm^{-1})**	λ_{max}^{calcd} (nm)	***f*** (oscillator strength)	Dominant orbital pair
**8**	450	21,500	453	0.6684	HOMO→LUMO
	350	4,800	369	0.1492	HOMO→LUMO+1
	289	15,900	306	0.1087	HOMO−1→LUMO
			298	0.3092	HOMO→LUMO+2
**9**	456	28,600	464	0.7459	HOMO→LUMO
	306	18,000	372	0.1557	HOMO→LUMO+1
			309	0.3280	HOMO→LUMO+2
**10**	457	12,500	459	0.8550	HOMO→LUMO
	321	12,500	371	0.1965	HOMO→LUMO+1
			327	0.2858	HOMO−1→LUMO
			317	0.2465	HOMO→LUMO+2
**11**	468	42,800	467	1.6806	HOMO→LUMO
			448	0.0138	HOMO→LUMO+1
	310	21,900	372	0.2733	HOMO→LUMO+2
			328	0.2369	HOMO−2→LUMO
**12**	487	49,900	496	1.8878	HOMO→LUMO
			458	0.0410	HOMO→LUMO+1
	354	18,000	379	0.1802	HOMO→LUMO+2
**13**	466	58,900	466	1.9158	HOMO→LUMO
					HOMO→LUMO+1
			454	0.0129	HOMO−1→LUMO
	344	34,300	373	0.3462	HOMO→LUMO+2

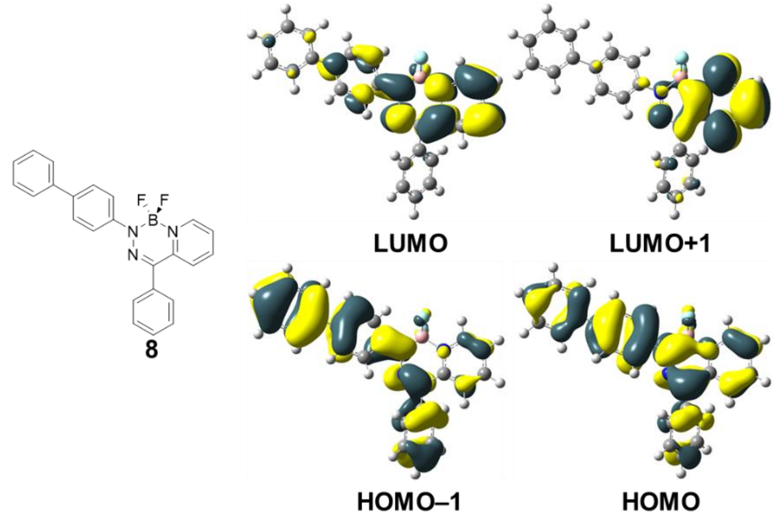

Figure S21. Molecular orbitals for BODIHY **8** (ground-state, LC-ωhPBE(ω=0.14)/DGDZVP2 SCRF=(PCM, Solvent=THF) method).

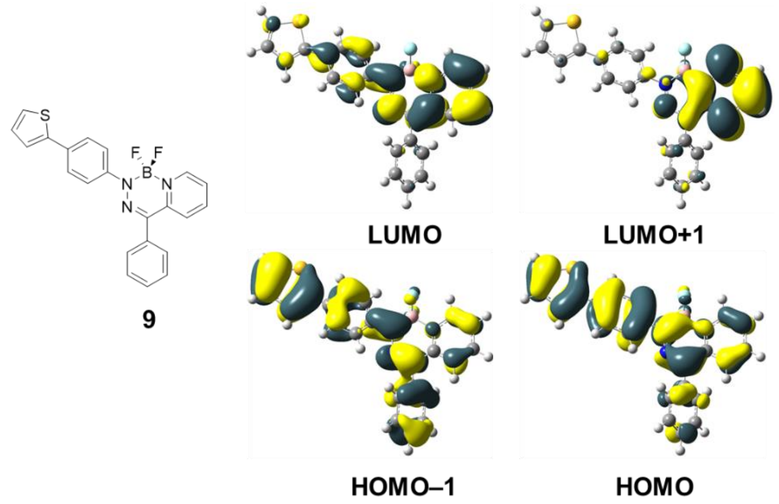

Figure S22. Molecular orbitals for BODIHY **8** (ground-state, LC-ωhPBE(ω=0.14)/DGDZVP2 SCRF=(PCM, Solvent=THF) method).

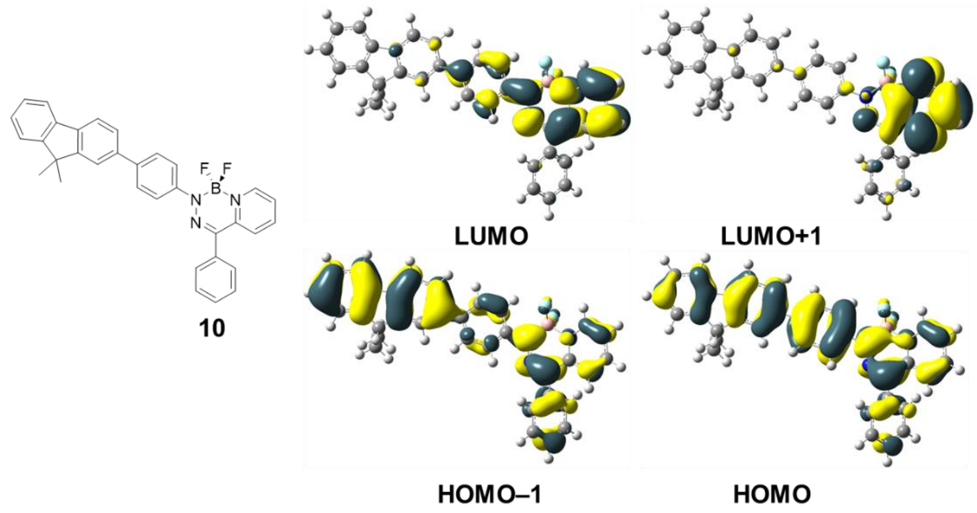

Figure S23. Molecular orbitals for BODIHY **10** (ground-state, LC-ωhPBE(ω=0.14)/DGDZVP2 SCRF=(PCM, Solvent=THF) method). The hexyl chains of BODIHY **10** were approximated as methyl groups.

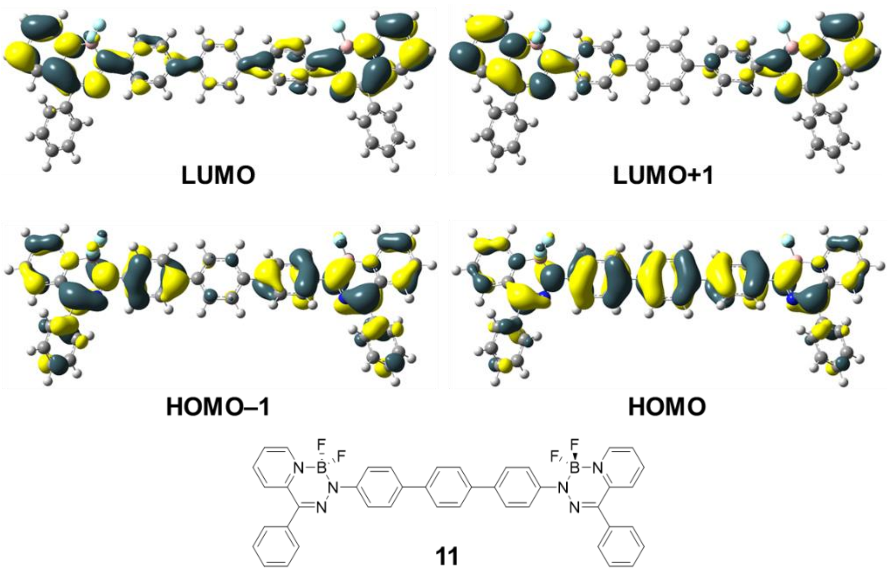

Figure S24. Molecular orbitals for BODIHY **11** (ground-state, LC-ωhPBE(ω=0.14)/DGDZVP2 SCRF=(PCM, Solvent=THF) method).

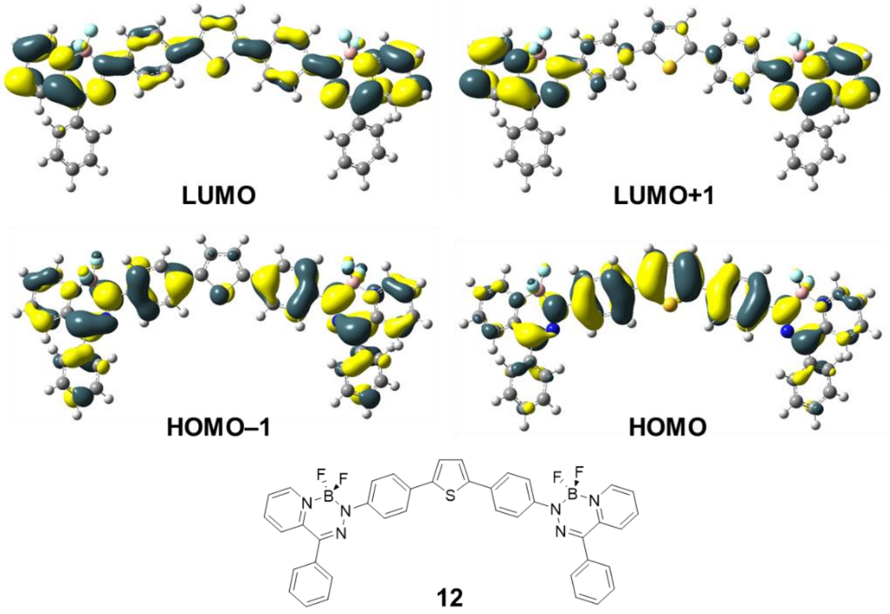

Figure S25. Molecular orbitals for BODIHY **12** (ground-state, LC-ωhPBE(ω=0.14)/DGDZVP2 SCRF=(PCM, Solvent=THF) method).

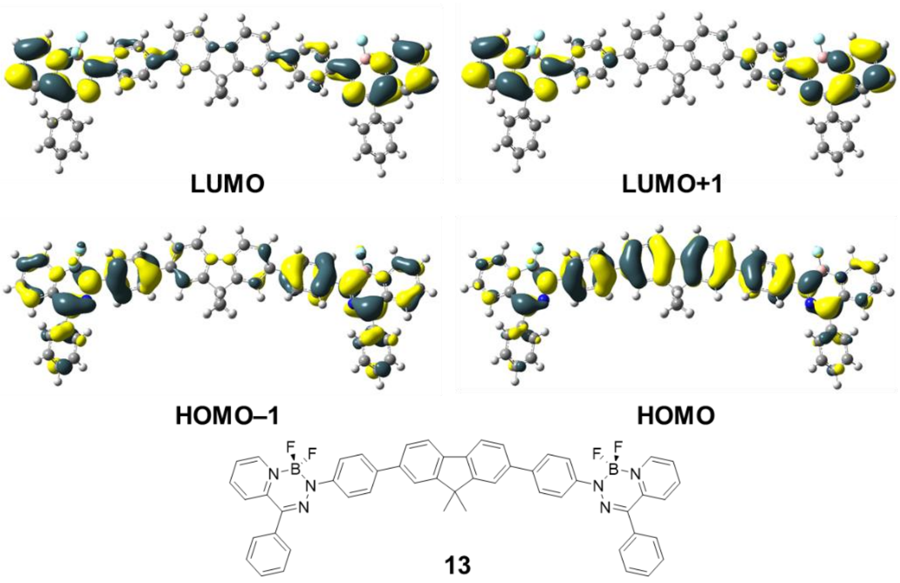

Figure S26. Molecular orbitals for BODIHY **13** (ground-state, LC-ωhPBE(ω=0.14)/DGDZVP2 SCRF=(PCM, Solvent=THF) method). The hexyl chains of BODIHY **13** were approximated as methyl groups.

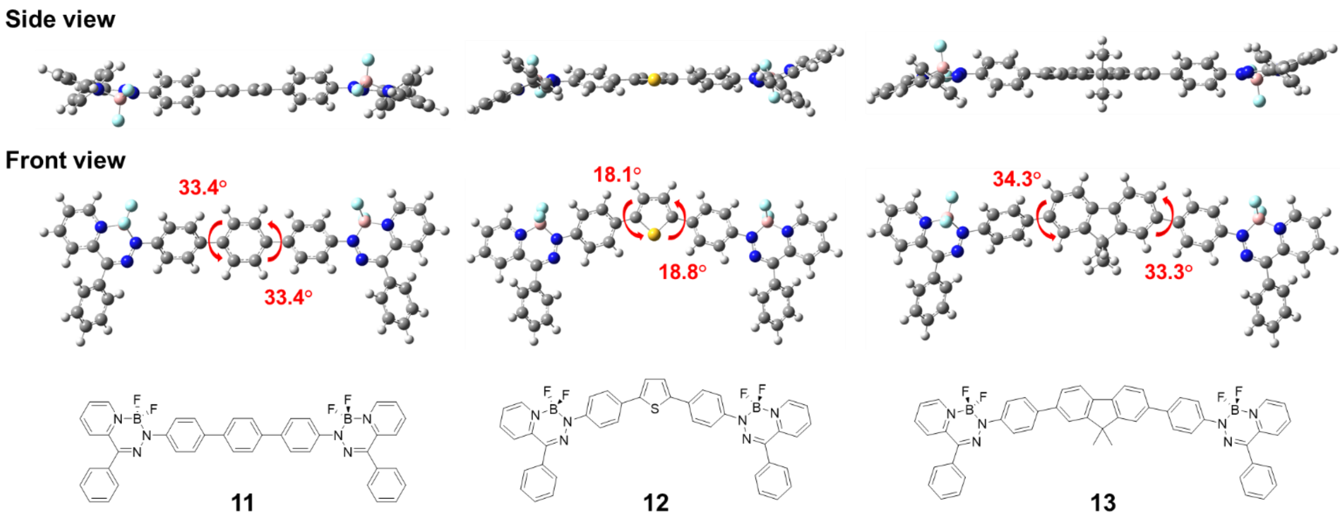

Figure S27. Angles between the planes defined by the *N*-aryl substituent on the BODIHY unit and the plane defined by the neighbouring aryl rings of the donor units. Calculated for the ground-state geometries using the LC-ωhPBE(ω=0.14)/DGDZVP2 SCRF=(PCM, Solvent=THF) method.

Optimized LC-ωhPBE(ω=0.14)/DGDZVP2 geometries of compounds **8**–**13** in THF solution (coordinates)

```
# LC-whPBE DGDZVP2 IOp(3/107=0140000000,3/108=0000001400)
  SCRF (PCM,Solvent=Tetrahydrofuran)
```

Compound **8**, ground-state structure (C_1 symmetry)
SCF Done: E(RLC-wHPBE) = -1313.38100944

```
 0,1
 C       3.24937    -0.81366    -0.20335
 C       2.53367     0.43088     0.03318
 H       2.47648    -4.05072    -0.29702
 C       3.12120    -3.17825    -0.40605
 C       4.59300    -0.86123    -0.64535
 C       5.18619    -2.08015    -0.95497
 C       4.43591    -3.26921    -0.83002
 H       5.14304     0.07269    -0.75674
 H       6.22192    -2.11375    -1.30088
 H       4.86462    -4.24500    -1.06107
 C       3.25637     1.73533     0.08062
 C       2.78948     2.83016    -0.67550
 C       4.38028     1.91359     0.91468
```

C	3.43603	4.07295	-0.60610
H	1.91588	2.69880	-1.31859
C	5.02686	3.15764	0.98423
H	4.73737	1.08466	1.53208
C	4.55906	4.24148	0.22249
H	3.06559	4.91155	-1.20194
H	5.89105	3.28334	1.64204
H	5.06304	5.20999	0.27649
C	-0.93485	-0.38023	0.09905
C	-1.45117	0.76804	-0.53248
C	-1.83037	-1.29738	0.68304
C	-2.83159	0.98973	-0.57691
H	-0.76274	1.47684	-0.99529
C	-3.20977	-1.07029	0.62259
H	-1.45151	-2.17767	1.20465
H	-3.20674	1.87421	-1.09882
H	-3.88084	-1.78281	1.10955
N	2.54572	-1.98340	-0.10093
N	1.21480	0.50226	0.10806
N	0.47066	-0.60948	0.14734
B	1.06591	-1.99529	0.44566
F	1.11871	-2.25018	1.84661
F	0.36301	-3.03558	-0.20537
C	-3.74320	0.07536	-0.00570
C	-5.21092	0.31053	-0.06512
C	-6.11376	-0.76821	-0.19302
C	-5.73960	1.61842	0.00648
C	-7.49838	-0.54778	-0.24740
H	-5.72867	-1.78820	-0.27549
C	-7.12387	1.84105	-0.04827
H	-5.06365	2.46861	0.13192
H	-8.17858	-1.39721	-0.35513
H	-7.51177	2.86134	0.01789
C	-8.01125	0.75870	-0.17554
H	-9.08988	0.93133	-0.21791

Compound **9**, ground-state structure (C_1 symmetry)
SCF Done: E(RLC-wHPBE) = -1634.06556361

0,1

C	3.25535	-0.85670	-0.20064
C	2.57778	0.40841	0.03966
H	2.38121	-4.06669	-0.32796
C	3.05470	-3.21437	-0.42172
C	4.60243	-0.94420	-0.62539

C	5.16083	-2.17941	-0.93543
C	4.37162	-3.34466	-0.82825
H	5.18330	-0.02757	-0.72297
H	6.19951	-2.24373	-1.26786
H	4.77224	-4.33218	-1.05982
C	3.34086	1.68922	0.10009
C	2.91479	2.80092	-0.65554
C	4.46207	1.82931	0.94497
C	3.59847	4.02300	-0.57486
H	2.04374	2.69891	-1.30740
C	5.14585	3.05264	1.02575
H	4.78793	0.98738	1.56205
C	4.71845	4.15349	0.26462
H	3.25949	4.87499	-1.17045
H	6.00745	3.14904	1.69184
H	5.25144	5.10581	0.32738
C	-0.91545	-0.28664	0.08583
C	-1.39039	0.90022	-0.50789
C	-1.84567	-1.19356	0.63065
C	-2.76072	1.16839	-0.55915
H	-0.67583	1.60306	-0.93880
C	-3.21539	-0.91641	0.57557
H	-1.50231	-2.10741	1.11754
H	-3.10229	2.08134	-1.05317
H	-3.91161	-1.62727	1.02988
N	2.51319	-2.00373	-0.11655
N	1.26198	0.52186	0.10979
N	0.48046	-0.56457	0.13236
B	1.02487	-1.97647	0.40594
F	1.04442	-2.26781	1.80040
F	0.29828	-2.97633	-0.28227
C	-3.70630	0.26713	-0.01832
C	-5.14680	0.56762	-0.08088
C	-5.76931	1.79854	-0.23139
S	-6.34593	-0.70087	0.02448
C	-7.19694	1.71531	-0.25329
H	-5.22036	2.73899	-0.29759
C	-7.65549	0.41943	-0.12225
H	-7.85819	2.57727	-0.35493
H	-8.68352	0.06078	-0.10438

Compound **10**, ground-state structure (C_1 symmetry)
SCF Done: E(RLC-wHPBE) = -1660.89636149

0,1

C	5.62321	-0.52388	-0.21487
C	4.71330	0.58699	0.01910
H	5.39336	-3.84453	-0.28694
C	5.88502	-2.87848	-0.40444
C	6.95424	-0.35240	-0.66441
C	7.73848	-1.45895	-0.97096
C	7.19465	-2.75449	-0.83565
H	7.34256	0.65859	-0.78362
H	8.76401	-1.32380	-1.32241
H	7.77684	-3.64788	-1.06419
C	5.21117	1.99316	0.05187
C	4.56895	2.98859	-0.71302
C	6.29276	2.36210	0.87932
C	5.00288	4.32132	-0.65862
H	3.72735	2.70894	-1.35141
C	6.72635	3.69611	0.93406
H	6.78281	1.60933	1.50311
C	6.08512	4.68047	0.16355
H	4.49845	5.08146	-1.26131
H	7.55990	3.96879	1.58686
H	6.42352	5.71904	0.20577
C	1.42577	-0.78333	0.11649
C	0.72279	0.26241	-0.51317
C	0.69681	-1.83357	0.70814
C	-0.67548	0.25363	-0.54747
H	1.28179	1.07356	-0.98229
C	-0.70142	-1.83574	0.65857
H	1.21856	-2.63873	1.22777
H	-1.19409	1.06287	-1.06862
H	-1.24234	-2.64751	1.15200
N	5.12228	-1.79282	-0.10203
N	3.40122	0.44046	0.10375
N	2.85035	-0.77813	0.15462
B	3.66781	-2.04532	0.45447
F	3.77080	-2.28035	1.85625
F	3.14258	-3.19077	-0.18706
C	-1.42116	-0.79625	0.03153
C	-2.90707	-0.80377	-0.01598
C	-3.61711	-2.02195	-0.13636
C	-3.63708	0.40613	0.05512
C	-5.01835	-2.05109	-0.18431
H	-3.05983	-2.95897	-0.21952
C	-5.03136	0.38446	0.00813
H	-3.10044	1.35204	0.17547
C	-5.72566	-0.84142	-0.11101

H	-5.54212	-3.00585	-0.28533
C	-6.00560	1.56002	0.08270
C	-7.17095	-0.55843	-0.13065
C	-7.35026	0.84070	-0.01862
C	-8.27452	-1.42008	-0.23564
C	-8.63730	1.38748	-0.01134
C	-9.56631	-0.86583	-0.22809
H	-8.13956	-2.50183	-0.32203
C	-9.74711	0.52687	-0.11689
H	-8.79139	2.46726	0.07500
H	-10.43946	-1.51915	-0.30905
H	-10.75891	0.94147	-0.11236
C	-5.80092	2.53671	-1.09623
H	-4.80501	3.00352	-1.04106
H	-5.88793	2.01569	-2.06164
H	-6.55357	3.34003	-1.06552
C	-5.87297	2.31533	1.42360
H	-6.01643	1.63531	2.27681
H	-4.87603	2.77522	1.50951
H	-6.62358	3.11849	1.48862
C	5.19406	10.05933	-2.37932

Compound **11**, ground-state structure (C_2 symmetry)
SCF Done: E(RLC-wHPBE) = -2394.70795977

0,1

C	0.39163	9.89248	-0.38848
C	0.41705	9.01860	0.75287
H	0.73012	9.53419	-3.66896
C	0.52309	10.07287	-2.75728
C	0.00520	11.24689	-0.32733
C	-0.10145	11.99296	-1.48155
C	0.16420	11.39799	-2.72560
H	-0.22396	11.67451	0.63644
H	-0.40128	13.03113	-1.42814
H	0.08758	11.95708	-3.64596
C	0.28506	9.54699	2.12842
C	-0.64902	8.98944	3.01016
C	1.11412	10.57917	2.58881
C	-0.75912	9.45857	4.31566
H	-1.27925	8.18167	2.66186
C	1.00003	11.05158	3.89429
H	1.87033	10.99277	1.93172
C	0.06099	10.49499	4.76099
H	-1.48706	9.01758	4.98509

H	1.65481	11.84260	4.23808
H	-0.02586	10.86027	5.77643
C	0.50410	5.69312	-0.53636
C	-0.30274	5.05764	0.41176
C	1.19470	4.92304	-1.47768
C	-0.41734	3.67487	0.41458
H	-0.83674	5.65599	1.13554
C	1.06525	3.54091	-1.46928
H	1.84003	5.39927	-2.20095
H	-1.06932	3.20049	1.13772
H	1.62815	2.95759	-2.18720
N	0.64012	9.34337	-1.61701
N	0.44197	7.70058	0.63974
N	0.63230	7.10659	-0.53947
B	1.13841	7.87141	-1.75066
F	2.56761	7.89342	-1.79830
F	0.63606	7.35830	-2.97896
C	0.25986	2.88504	-0.52696
C	0.12761	1.41434	-0.52642
C	0.06099	0.69200	-1.72687
C	0.06370	0.69175	0.67406
C	-0.06099	-0.69200	-1.72687
H	0.09091	1.22418	-2.66992
C	-0.06370	-0.69175	0.67406
H	0.12997	1.22045	1.61730
C	-0.12761	-1.41434	-0.52642
H	-0.09091	-1.22418	-2.66992
H	-0.12997	-1.22045	1.61730
C	-0.25986	-2.88504	-0.52696
C	0.41734	-3.67487	0.41458
C	-1.06525	-3.54091	-1.46928
C	0.30274	-5.05764	0.41176
H	1.06932	-3.20049	1.13772
C	-1.19470	-4.92304	-1.47768
H	-1.62815	-2.95759	-2.18720
C	-0.50410	-5.69312	-0.53636
H	0.83674	-5.65599	1.13554
H	-1.84003	-5.39927	-2.20095
N	-0.63230	-7.10659	-0.53947
N	-0.44197	-7.70058	0.63974
B	-1.13841	-7.87141	-1.75066
C	-0.41705	-9.01860	0.75287
N	-0.64012	-9.34337	-1.61701
F	-2.56761	-7.89342	-1.79830
F	-0.63606	-7.35830	-2.97896

C	-0.39163	-9.89248	-0.38848
C	-0.28506	-9.54699	2.12842
C	-0.52309	-10.07287	-2.75728
C	-0.00520	-11.24689	-0.32733
C	0.64902	-8.98944	3.01016
C	-1.11412	-10.57917	2.58881
H	-0.73012	-9.53419	-3.66896
C	-0.16420	-11.39799	-2.72560
C	0.10145	-11.99296	-1.48155
H	0.22396	-11.67451	0.63644
C	0.75912	-9.45857	4.31566
H	1.27925	-8.18167	2.66186
C	-1.00003	-11.05158	3.89429
H	-1.87033	-10.99277	1.93172
H	-0.08758	-11.95708	-3.64596
H	0.40128	-13.03113	-1.42814
C	-0.06099	-10.49499	4.76099
H	1.48706	-9.01758	4.98509
H	-1.65481	-11.84260	4.23808
H	0.02586	-10.86027	5.77643
C	4.45009	-0.65526	-0.46863

Compound **12**, ground-state structure (C_2 symmetry)
SCF Done: E(RLC-wHPBE) = -2715.40017193

0,1

C	-0.40022	9.41505	-0.34393
C	-0.76768	8.24892	-1.13296
H	0.22593	10.04539	2.86452
C	0.15938	10.27326	1.80041
C	-0.05090	10.65968	-0.91964
C	0.39410	11.70338	-0.11564
C	0.49972	11.50956	1.27845
H	-0.11821	10.77607	-2.00088
H	0.66801	12.66171	-0.56296
H	0.84288	12.30274	1.94358
C	-1.05640	8.36986	-2.59180
C	-0.43470	7.49913	-3.51028
C	-1.98660	9.31566	-3.07225
C	-0.72973	7.57710	-4.87932
H	0.28244	6.76112	-3.14274
C	-2.28212	9.39289	-4.44239
H	-2.50156	9.97815	-2.37095
C	-1.65319	8.52595	-5.35161
H	-0.23610	6.89863	-5.58037

H	-3.01202	10.12458	-4.79902
H	-1.88305	8.58674	-6.41857
C	-0.46971	5.42369	1.01501
C	0.00000	4.47863	0.08194
C	-0.83018	4.98368	2.30491
C	0.11192	3.13108	0.43516
H	0.28308	4.81118	-0.91767
C	-0.71372	3.63447	2.64989
H	-1.22429	5.69112	3.03592
H	0.50204	2.42604	-0.30480
H	-1.02835	3.31950	3.64761
N	-0.27900	9.25588	1.01018
N	-0.76068	7.01799	-0.64915
N	-0.58269	6.79769	0.65949
B	-0.71549	7.91171	1.71142
F	-2.06482	8.06156	2.14346
F	0.12640	7.70342	2.82818
C	-0.23654	2.67583	1.72699
C	-0.10981	1.26234	2.11303
C	-0.06295	0.70948	3.38507
S	0.00000	0.00000	0.90977
C	0.06295	-0.70948	3.38507
H	-0.09985	1.30820	4.29636
C	0.10981	-1.26234	2.11303
H	0.09985	-1.30820	4.29636
C	0.23654	-2.67583	1.72699
C	-0.11192	-3.13108	0.43516
C	0.71372	-3.63447	2.64989
C	0.00000	-4.47863	0.08194
H	-0.50204	-2.42604	-0.30480
C	0.83018	-4.98368	2.30491
H	1.02835	-3.31950	3.64761
C	0.46971	-5.42369	1.01501
H	-0.28308	-4.81118	-0.91767
H	1.22429	-5.69112	3.03592
N	0.58269	-6.79769	0.65949
N	0.76068	-7.01799	-0.64915
B	0.71549	-7.91171	1.71142
C	0.76768	-8.24892	-1.13296
N	0.27900	-9.25588	1.01018
F	2.06482	-8.06156	2.14346
F	-0.12640	-7.70342	2.82818
C	0.40022	-9.41505	-0.34393
C	1.05640	-8.36986	-2.59180
C	-0.15938	-10.27326	1.80041

C	0.05090	-10.65968	-0.91964
C	0.43470	-7.49913	-3.51028
C	1.98660	-9.31566	-3.07225
H	-0.22593	-10.04539	2.86452
C	-0.49972	-11.50956	1.27845
C	-0.39410	-11.70338	-0.11564
H	0.11821	-10.77607	-2.00088
C	0.72973	-7.57710	-4.87932
H	-0.28244	-6.76112	-3.14274
C	2.28212	-9.39289	-4.44239
H	2.50156	-9.97815	-2.37095
H	-0.84288	-12.30274	1.94358
H	-0.66801	-12.66171	-0.56296
C	1.65319	-8.52595	-5.35161
H	0.23610	-6.89863	-5.58037
H	3.01202	-10.12458	-4.79902
H	1.88305	-8.58674	-6.41857
C	7.28792	-1.29730	-1.11835

Compound **13**, ground-state structure (C_2 symmetry)
SCF Done: E(RLC-wHPBE) = -2742.23076751

0,1

C	0.87619	11.77623	0.30528
C	0.94206	10.65775	1.23333
H	1.07582	12.17552	-2.99407
C	0.91179	12.48754	-1.96234
C	0.51330	13.08680	0.69757
C	0.36283	14.08736	-0.25621
C	0.56919	13.78425	-1.61933
H	0.33564	13.28925	1.75334
H	0.07777	15.09699	0.04866
H	0.46220	14.54045	-2.39780
C	0.89336	10.88011	2.70784
C	0.00000	10.13662	3.50626
C	1.76694	11.79428	3.33379
C	-0.02536	10.30834	4.89813
H	-0.67494	9.42353	3.02675
C	1.74173	11.96543	4.72688
H	2.48753	12.35703	2.73380
C	0.84406	11.22521	5.51432
H	-0.72701	9.72846	5.50386
H	2.43098	12.67073	5.19891
H	0.82421	11.35920	6.59902
C	0.89068	7.69100	-0.73830

C	0.10505	6.87389	0.09787
C	1.51906	7.11343	-1.85897
C	-0.04434	5.51195	-0.18320
H	-0.39306	7.31792	0.96122
C	1.35421	5.75166	-2.13451
H	2.15614	7.72045	-2.50417
H	-0.68395	4.90811	0.46615
H	1.87816	5.32343	-2.99308
N	1.06178	11.51234	-1.02514
N	0.93370	9.39380	0.84219
N	1.04734	9.07837	-0.45348
B	1.52916	10.08571	-1.51023
F	2.95084	10.10035	-1.60617
F	0.96376	9.84478	-2.78336
C	0.57142	4.91855	-1.30681
C	0.40341	3.47152	-1.60324
C	0.35248	3.01140	-2.94057
C	0.28847	2.52922	-0.55394
C	0.19390	1.65103	-3.24166
H	0.41249	3.73589	-3.75723
C	0.13052	1.17443	-0.84595
H	0.35264	2.86872	0.48422
C	0.08349	0.73084	-2.18798
H	0.14972	1.32341	-4.28413
C	0.00000	0.00000	0.12370
C	-0.08349	-0.73084	-2.18798
C	-0.13052	-1.17443	-0.84595
C	-0.19390	-1.65103	-3.24166
C	-0.28847	-2.52922	-0.55394
C	-0.35248	-3.01140	-2.94057
H	-0.14972	-1.32341	-4.28413
C	-0.40341	-3.47152	-1.60324
H	-0.35264	-2.86872	0.48422
H	-0.41249	-3.73589	-3.75723
C	-1.25746	0.14084	1.00938
H	-1.17358	1.02687	1.65797
H	-2.16479	0.24387	0.39526
H	-1.37412	-0.74261	1.65635
C	1.25746	-0.14084	1.00938
H	2.16479	-0.24387	0.39526
H	1.37412	0.74261	1.65635
H	1.17358	-1.02687	1.65797
C	-0.57142	-4.91855	-1.30681
C	0.04434	-5.51195	-0.18320
C	-1.35421	-5.75166	-2.13451

C	-0.10505	-6.87389	0.09787
H	0.68395	-4.90811	0.46615
C	-1.51906	-7.11343	-1.85897
H	-1.87816	-5.32343	-2.99308
C	-0.89068	-7.69100	-0.73830
H	0.39306	-7.31792	0.96122
H	-2.15614	-7.72045	-2.50417
N	-1.04734	-9.07837	-0.45348
N	-0.93370	-9.39380	0.84219
B	-1.52916	-10.08571	-1.51023
C	-0.94206	-10.65775	1.23333
N	-1.06178	-11.51234	-1.02514
F	-2.95084	-10.10035	-1.60617
F	-0.96376	-9.84478	-2.78336
C	-0.87619	-11.77623	0.30528
C	-0.89336	-10.88011	2.70784
C	-0.91179	-12.48754	-1.96234
C	-0.51330	-13.08680	0.69757
C	0.00000	-10.13662	3.50626
C	-1.76694	-11.79428	3.33379
H	-1.07582	-12.17552	-2.99407
C	-0.56919	-13.78425	-1.61933
C	-0.36283	-14.08736	-0.25621
H	-0.33564	-13.28925	1.75334
C	0.02536	-10.30834	4.89813
H	0.67494	-9.42353	3.02675
C	-1.74173	-11.96543	4.72688
H	-2.48753	-12.35703	2.73380
H	-0.46220	-14.54045	-2.39780
H	-0.07777	-15.09699	0.04866
C	-0.84406	-11.22521	5.51432
H	0.72701	-9.72846	5.50386
H	-2.43098	-12.67073	5.19891
H	-0.82421	-11.35920	6.59902
